# *Cul3* Postnatal Homozygous Deletion in Forebrain Results in Behavioral Differences

**DOI:** 10.1111/gbb.70039

**Published:** 2025-10-20

**Authors:** Prathibha Sekar, Qiang‐qiang Xia, Alex C. Stokes, Feiyu Quan, Jeffrey D. Singer, Craig M. Powell

**Affiliations:** ^1^ Department of Neurobiology and Civitan International Research Center for Neurodevelopmental Disorders University of Alabama at Birmingham Birmingham Alabama USA; ^2^ Department of Biology Portland State University Portland Oregon USA

**Keywords:** autism, *CaMKIIα*, *Cullin 3 (Cul3)*, delayed postnatal deletion

## Abstract

Large‐scale human genetic studies implicate multiple genes that regulate protein ubiquitination in autism spectrum disorder (ASD). De novo loss‐of‐function mutations in the gene *CULLIN3 (CUL3)* are implicated in autism and intellectual disability (ID). CUL3 is an essential component of an E3 ubiquitin ligase complex required for ubiquitination of substrates, often a signal for proteasomal degradation. Homozygous deletion of *Cul3* is embryonically lethal. Recent studies show heterozygous deletion of *Cul3* results in phenotypes with some face validity for autism in constitutive and conditional *Cul3* heterozygotes. To understand the function of *Cul3* in late postnatal development and function in the brain, we crossed mice expressing Cre‐recombinase under the control of the *CaMKIIα* promoter with conditional (floxed) *Cul3* mice that resulted in viable homozygotes. In this study, we demonstrate that delayed postnatal deletion of *Cul3* in predominantly forebrain excitatory neurons leads to robust behavioral differences across multiple behaviors. *Cul3* conditional homozygotes show repetitive jumping, reduced marble burying, increased locomotion, impaired motor coordination, and increased hindlimb clasping. We were successfully able to replicate most of these findings in an independent cohort. Our future studies are aimed at gaining mechanistic insights into *Cul3* function in the adult brain.

## Graphical abstract

To understand the function of *Cul3*, a high‐risk autism gene, we generated a delayed postnatal deletion mouse model in predominantly forebrain excitatory neurons, which resulted in viable homozygotes. We tested them on multiple autism‐relevant behaviors and found behavioral differences: Cul3 homozygotes showed decreased motor coordination, decreased marble burying, increased locomotor activity, increased repetitive jumping, and increased hindlimb clasping.

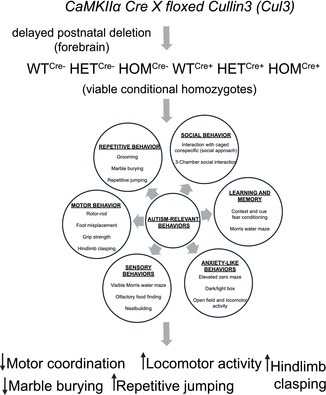

## 1 Introduction

Autism spectrum disorder (ASD) is a complex, heterogeneous, and sometimes debilitating neurodevelopmental disorder (NDD) characterized by impairments in social communication and interaction, and restricted, repetitive behaviors [1]. Pathogenic genetic variants are major causes of ASD [2]. Several human sequencing studies have identified de novo loss‐of‐function mutations in *Cul3* associated with ASD with an FDR < 0.01 [3, 4, 5, 6, 7]. More recently, patients with primarily de novo missense, nonsense, splice‐site, or frameshift variants in *CUL3* show developmental delay, intellectual disability (ID), and behavioral abnormalities [8].

CUL3 is a scaffolding protein that is part of a CUL3‐RING‐E3 ubiquitin ligase (CRL) complex essential for ubiquitination of substrates, often leading to their proteasomal degradation [9, 10]. Specificity for substrate recognition is achieved by adaptor proteins with BTB domains (broad‐complex [Br‐C], tramtrack [ttk], bric à brac [bab]) that bind to the N‐terminus of CUL3. RING‐Box protein1 (RBX1) binds to the C‐terminal domain of CUL3 and recruits E2 ligase to facilitate the transfer of ubiquitin to the substrate. CUL3 is expressed ubiquitously in all cells through embryonic development and postnatally in humans [11] and mice [12, 13, 14]. More specifically, in mice, protein expression of CUL3 peaks embryonically between embryonic day 14 (E14) and postnatal day 21 (P21), though it is continuously expressed in adulthood [13].

Complete *Cul3* deletion is embryonically lethal at approximately E7.5 [15]. This finding is consistent with our own observations in our study of constitutive *Cul3* mice [16]. Indeed, rodent models with cell‐type‐specific, early embryonic conditional deletion of *Cul3* (from roughly E11.5—*Emx‐Cre* [13, 17], *E11.5—Nex‐Cre* [18], E13.5—*GFAP* [18], E12.5—*ChAT‐Cre E12.5* [19]), leads to early postnatal lethality. Interestingly, in a study using a tamoxifen‐regulated cre‐recombinase to cause whole‐body knockout, homozygous *Cul3* deletion at P30–P40 led to lethality ~20 days after tamoxifen injections [13]. However, a cell‐type‐specific DAT promoter line that drives cre‐recombinase expression in dopaminergic cells led to viable homozygotes when crossed with *Cul3* floxed mice that survived into adulthood [20]. Interestingly, Cre expression driven by the DAT promoter begins ~E15, suggesting *Cul3* deletion in certain brain cell types may not lead to early lethality.

To better understand its function in the brain, recent studies show haploinsufficiency in constitutive and conditional cell‐type specific, brain‐region specific *Cul3 deletion* may affect brain morphology [13, 16], neuronal migration [13], synaptic transmission [12, 13, 16], and behaviors with some face validity for ASD [12, 16, 17, 20].

In this study, we generated a forebrain‐specific, postnatal deletion of *Cul3* in predominantly excitatory neurons that survived into adulthood in the homozygotes. Using this model, we sought to understand the effect of homozygous deletion of *Cul3* across many behavioral outcome measures as a starting point to better understand the function of *Cul3* postnatally.

## 2 Methods

All animal procedures were in accordance with the Care and Use of Laboratory Animals and were approved by the Institutional Animal Care and Use Committee at the University of Alabama at Birmingham. Mice were housed in temperature‐controlled, ventilated cages with ad libitum access to food and water on a 12 h dark/light cycle.

### 2.1 Generation of Mice

Homozygous floxed *Cul3* mice (on a C57BL/6J background), a gift from Dr. Jeffrey D. Singer (Portland State University, Oregon), were crossed with transgenic mice expressing cre recombinase driven by a *CaMKII* promoter (obtained from Jackson laboratory, strain—B6.Cg‐Tg [Camk2a‐cre] T29‐1Stl, Stock #005359). Cre‐positive, floxed *Cul3* heterozygotes were further crossed with Cre‐negative, floxed *Cul3* heterozygotes to generate cre‐positive and cre‐negative, conditional *Cul3* homozygotes (HOM^Cre+^ and HOM^Cre−^), conditional heterozygotes (HET^Cre+^ and HET^Cre−^), and wild‐type controls, respectively (WT^Cre+^ and WT^Cre−^). For immunoblot experiments to assess protein expression, male adult mice (P90: WT^Cre+^ = 6, HET^Cre+^ = 6, HOM^Cre+^ = 6) were used. A detailed break‐down of mice used for behavioral assays is provided below.

### 2.2 Genotyping

Tail and toe biopsies were collected from P7 pups and digested in 50 mM NaOH (~200 μL), followed by heat treatment at 95°C for a period of 30–40 min. After which, 1 M Tris–HCl (~17 μL) was added to neutralize the tail extract. Qualitative polymerase chain reaction (PCR) was run with crude DNA extract. PCR products were visualized on 1.5% agarose gels containing SYBR Safe DNA gel stain (Thermo Fisher, Scientific Catalog #S33102) using LICOR Odyssey Fc. The following primers were used to identify genotypes: *Cre‐recombinase*—forward 5′‐GCGGTCTGGCAGTAAAAACTATC‐3′, reverse 5′‐GTGAAACAGCATTGCTGTCACTT‐3′; internal positive control (IPC; interleukin gene 2—*Il2*)—forward 5′‐CTAGGCCACAGAATTGAAAGATCT‐3′, reverse 5′‐GTAGGTGGAAATTCTAGCATCATCC‐3′; *Cul3* WT forward 5′‐TGCTCTCTCTGGGTGATCCT‐3′, reverse 5′‐ACACCATGGACTATGAACATGC‐3′; *Cul3* floxed forward 5′‐AGCCAACGCTATGTCCTGAT‐3′, reverse 5′‐CTGAATGAACTGCAGGACGA‐3′. Bands were identified for the following PCR products as follows—*Cre‐recombinase*—100 bp, IPC—324 bp, *Cul3* WT—466 bp, *Cul3* floxed—504 bp. IPC primers were added to Cre‐recombinase PCR reactions to ensure the integrity of the DNA.

### 2.3 Tissue Isolation and Homogenization

Mice were briefly anesthetized under isoflurane and rapidly decapitated, following which the brain was rapidly dissected. Fresh tissue (hippocampi and cortex) was lysed in ice‐cold RIPA buffer (Thermo Fisher Scientific, Catalog #89901) supplemented with protease and phosphatase inhibitors (Halt Protease and Phosphatase inhibitors, Thermo Fisher Scientific, Catalog #1861281). Individual samples were sonicated, centrifuged at 4°C for 1 min at 1000 rpm and the supernatant isolated. A detergent compatible BCA assay (Pierce Micro BCA protein assay kit, Thermo Scientific, Catalog #23235) was performed to determine protein concentrations. 2× Laemmli sample buffer (Bio‐Rad, Catalog #1610737) with 2‐mercaptoethanol (Bio‐Rad, Catalog #1610710) was added to the sample and boiled for 5 min at 100°C.

### 2.4 Immunoblotting

Fifty micrograms of protein was loaded onto 4%–20% gradient SDS‐PAGE gels (Bio‐Rad, Catalog #5671094). After separation, a semidry transfer using Bio‐Rad Trans‐Blot Turbo transfer was done for 7 min onto a PVDF membrane (pore size 0.22 μm). Following this, the membrane was blocked in TBS (Intercept Blocking Buffer, LICORbio, Catalog #927‐60001) for 1–2 h at room temperature. This was followed by incubation of the primary antibody in blocking buffer (TBS) with 0.1% Tween at 4°C overnight. After incubation with corresponding secondary antibodies, membranes were imaged using LICOR Odyssey Fc. β‐Actin was used as the internal loading control. Immunoblots were analyzed using Quantity One (Version 4.6.6). Raw intensity values from CUL3 reactive bands were normalized to β‐actin intensity values, which were then normalized to the average of WT signals.

### 2.5 Antibodies

The following primary antibodies were used: mouse anti‐CUL3 (1:500, BD Biosciences, CAT#611848) and mouse anti‐β‐actin (1:1000, MP Biomedicals, CAT#691001). Secondary antibodies were as follows: goat antimouse‐680 (Thermo Scientific, CAT#35518).

### 2.6 Behavior

Mice that underwent behavior were group‐housed with littermates except before and during the nestbuilding assay (see below). All mice had ad libitum access to food and water throughout all experiments except before and during the olfactory food finding assay. To ensure overall well‐being, nestlets were part of the home‐cage environment. Behavioral experiments were performed during the light cycle (between 8 a.m. and 6 p.m.). Before testing on each day, mice were habituated for at least 30 min in the testing room. At least 1 day of rest was given in between assays (with the interval between assays ranging from a day to a month) to avoid potential confounds from previous behavior assays. All behavioral experiments were performed by an experimenter blind to genotypes.

Behavioral testing was performed on two separate cohorts. The first cohort consisted of 44 mice that were sex‐matched littermates drawn from 12 litters: WT^Cre+^ *n* = 19 (F = 10, M = 9), HET^Cre+^ *n* = 17 (F = 10, M = 7), and HOM^Cre+^ *n* = 8 (F = 2, M = 6). For the first cohort, the behavioral battery began when the mice were 3–5 months old. The second cohort consisted of 64 mice that were sex and age‐matched controls drawn from 17 litters; WT^Cre−^ *n* = 12 (F = 6, M = 6), HET^Cre−^ *n* = 12 (F = 7, M = 5), HOM^Cre−^ *n* = 10 (F = 5, M = 5), WT^Cre+^ *n* = 12 (F = 5, M = 7), HET^Cre+^ *n* = 10 (F = 5, M = 5), and HOM^Cre+^ *n* = 8 or 7 (F = 3 or 2, *M* = 8). Mice in the second cohort underwent behavioral testing beginning at 2 and a half months of age. Behavioral testing on the second cohort began approximately after 5 months after the conclusion of testing in the first cohort.

The order of behavioral testing for the first cohort was: elevated zero maze, dark–light box, open field, grooming, social approach, 3‐chamber social interaction, marble burying, rotarod, olfactory food finding, fear conditioning, Morris water maze, hindlimb clasping, visible Morris water maze, and nestbuilding.

For the second cohort, the order of behavioral testing was: elevated zero maze, dark–light box, open field, grooming, marble burying, grip strength, foot misplacement, rotarod, social approach, 3‐chamber social interaction, hindlimb clasping, Morris water maze, visible Morris water maze, and nestbuilding.

### 2.7 Acquisition of Data

Most behavioral data were acquired using Noldus Ethovision XT (Version 15 or 16) unless indicated otherwise. The Morris water maze in our first cohort was acquired using Ethovision XT Version 7. For fear conditioning, FreezeFrame software (Actimetrics) was used. Rotarod (Rotarod, Version 3, San Diego Instruments) and Foot misplacement apparatus (Locotronic, Bioseb) had their own respective software for data acquisition. Adjustments in behavioral settings between cohorts were made due to technical reasons including change in camera, distance of camera from the light source, change in location of the arena within the same testing room for better clarity of arena and subject. However, no drastic changes were made in the approach to the experiments unless noted otherwise.

*Elevated zero maze*: This assay was performed similar to previous descriptions with modifications [21]. In a dimly lit (~2 lx—first cohort and 7 lx—second cohort) room, mice were placed in the open arm (first cohort) and the interface between the open and closed arm (second cohort) on an elevated zero maze (which consists of two open arms and closed arms in a circular track which was 5 cm in width, the outer diameter of the circular track was 61 cm and inner diameter 49.5 cm). In the first cohort, mice were allowed 5 min to explore the maze. In the second cohort, mice were given 10 min of exposure time.

*Dark/light box*: This assay was performed similarly to previous descriptions with minor modifications [21]. In a brightly lit room (~12.5 lx—first cohort and ~30 lx—second cohort), mice were habituated in the dark side of the chamber (47 × 22 × 22 cm with the dark chamber being 19 × 22 × 22 cm and the door 8.5 × 5 cm) for 2 min, after which the door to the light side was removed. Mice were given 10 min to freely explore the dark and light chamber.

*Open field and locomotor activity*: Mice were placed in an open field arena (44 × 44 × 30 cm) in a dimly lit room (~3 lx—first cohort and 7 lx—second cohort). For our first cohort, mice were allowed to explore the open field for a total of 20 min. Time spent in the center was measured during the first 10 min [21] and locomotor activity was taken as the cumulative distance traveled during the entire 20 min exploration period. For the second cohort, time spent in the open field was increased to 60 min.

*Grooming*: Mice were habituated to a novel home‐cage (31 × 19.5 × 14 cm) without bedding for 10 min, after which they were scored for grooming behavior for a total of 10 min by an experimenter blind to genotype [21]. Rearing behavior was defined as mice licking or rubbing their face or any part of their body with either their forepaws or mouth.

*Marble burying*: In a novel cage (31 × 19.5 × 14 cm) with 5 cm fresh bedding, 20 marbles were arranged in a grid‐like fashion with roughly equal spacing. Mice were introduced and allowed to explore the cage for 30 min. The number of marbles buried was counted, with a marble considered buried if at least two‐third was buried [22].

*Grip strength*: Mice were placed on a metal grid connected to a force transducer (San Diego Instruments). Once they gripped the metal grid, they were gently pulled back, and the highest tensile force (N) was recorded. Each mouse was given a total of five pulls with 1 min between each pull. Five pulls were averaged and normalized to the body weight of the mouse [23].

*Foot misplacement*: This assay was performed as previously described with minor modifications [24]. The foot misplacement apparatus (Bioseb Instruments) consists of a horizontal ladder with 1‐cm spaced rods. Mice were placed at the start of the ladder where a bright white light is on, which acts as an aversive stimulus to motivate mice to traverse the length of the ladder to the dark end. A total of three trials per mouse was given with a maximum time to traverse the length of the ladder set at 1 min. If a mouse exceeded more than 1 min or turned around during the run, the trial was discarded.

*Rotarod*: Mice were placed on the stationary rod in a well‐lit room and the apparatus activated. Acceleration began at 4 rpm (first cohort) or 0 rpm (second cohort) with a maximum set to 45 rpm over 5 min. Each mouse was given a total of eight trials with four trials per day and 30‐min intervals between the training sessions [22].

*Olfactory food finding*: Mice were food‐restricted for 24 h prior to this assay. To acclimatize them to the food reward, a small portion of the food reward (Nutter Butter cookies) was introduced once into their home‐cage a day prior to the test during the food deprivation period. The following day (test day), the same food reward was buried ~1 cm below bedding in a novel cage (31 × 19.5 × 14 cm), and the latency to find the reward was recorded manually by an experimenter. The location of the reward varied from cage to cage [25].

*Caged conspecific test*: Mice were placed in an open field arena (44 × 44 × 30 cm) with an inverted pencil cup. For the first 5 min (inanimate approach), mice were allowed to explore the arena with an inverted empty pencil cup. For the next 5 min (social approach), a sex‐matched novel stimulus mouse was introduced in the inverted pencil cup and the experimental mouse was allowed to explore the arena. Interaction was defined as the center point of the mouse located in the interaction zone (~3–4 cm around the perimeter of the pencil cup) [22].

*3‐Chamber social interaction*: Mice were habituated to the 3‐chamber apparatus (60 × 40 × 22 cm divided into three compartments with two dividers placed 20 cm away from each edge along the length of the box of [20 × 40 cm] with a central 3.5 × 3.5 cm door) for 10 min. During the next 10 min (sociability or social vs. inanimate phase), an inanimate object (an inverted pencil cup) was placed in one chamber and a sex‐matched adult social target underneath an inverted pencil cup was placed in the opposite chamber (counterbalanced locations). In the final 10 min of the assay (preference for social novelty phase), the inanimate object was replaced with a novel sex‐matched adult social target. Interaction was defined as the center‐point in the interaction zone (~3–4 cm around the pencil cup). Social preference index was plotted as time spent with the social target/(total time spent with the inanimate object + social target) and novelty social preference index was plotted as time spent with the novel target/(total time spent with the familiar mouse + novel mouse) [22].

*Fear conditioning*: Fear conditioning was performed as previously described with minor modifications [22]. Mice were placed in a Plexiglas shock box with clear front and rear walls, and a steel grid as the floor (Coulbourn Instruments). After a 2‐min exploration period, three, 30‐s, ~90‐dB acoustic conditioned stimuli (white noise) coterminated with a 2 s, 0.5 mA foot shock, with 2‐min interstimulus intervals followed by a final 2 min in the box. Freezing behavior was measured by setting the threshold to 1 with a bout duration of 1 s. On the morning of the second day, for contextual fear testing mice were placed in the same context for 5 min and freezing was measured. Cued fear memory testing was performed in the afternoon of Day 2. Mice were placed in an altered context and allowed to explore the novel context for 3 min before playing the white noise for 3 min. The environmental context was altered by introducing different textured plastic walls into the shock box, placing a semitranslucent plastic plate in place of the steel grid, and adding vanilla extract as a novel olfactory cue.

*Morris water maze*: This assay was performed as previously described with minor modifications [22]. A white platform (9 in. in diameter) was submerged in a large pool (diameter ~120 cm) filled with water that was maintained at 22°C–23°C. The water was mixed with nontoxic white tempera paint. The room was decorated with spatial cues (high‐contrast white and black shapes). For both cohorts, mice were given a total of four trials per day with a minimum of ~1–2 min intertrial interval. Mice were introduced into the pool from four different locations for trials, and these locations were randomized across each day. A total of 60 s was given per trial to find the platform. If mice failed to find the platform, the experimenter gently guided the mice to the platform and was allowed to remain on the platform for 15 s before being removed from the pool. A total of 11 days (first cohort) or 12 days (second cohort) of training was followed by the probe trial during which the platform was removed, and mice were allowed to swim freely for 60 s. Two probe trials were performed in our second cohort (Days 8 and 14).

*Visible Morris water maze*: A visible cue (a 20 cc inverted syringe with black cubic Styrofoam attached to the top) was taped to the platform and mice were given a maximum of 1 min to find the platform. Three (first cohort) or four (second cohort) trials were given per mouse. If the mice did not find the platform, they were gently guided to the platform or were picked up by the tail and placed on the platform before removing them from the pool [22].

*Nestbuilding*: A modified version of this assay was performed based on previous studies [26, 27]. A day prior to the assay, mice were singly housed without any nestlets. The following morning, a nestlet 5 × 5 × 0.5 cm was introduced to the cage and 24 h later, the height and width of the nestlet were measured. For our second cohort, we videorecorded the first 90 min of the assay.

*Hindlimb clasping*: Clasping was assessed by gently lifting mice by their tail a few centimeters above their bedding for approximately 10 s. An experimenter, blinded to genotype, evaluated the mice for the clasping phenotype, which was defined as an attempt to draw their hindlimbs toward the midline of the torso. Mice that displayed any degree of clasping were given a score of 1 and mice that splayed their hindlimbs were given a score of 0. This scoring system was loosely based on a previous study [28].

### 2.8 Statistical Analyses

Data were plotted as mean ± SEM unless otherwise indicated. Statistical significance was set at *p* < 0.05 (two‐tailed tests; *p < 0.05, **p < 0.01, ***p < 0.001, ****p < 0.0001). Normality was visualized using Q–Q plots and frequency histograms, along with skewness and kurtosis values, and assessed using tests of normality (Shapiro–Wilk and Kolmogorov–Smirnov). Homogeneity of variance across groups was assessed using Levene's test and homoscedasticity plots. In some cases, where variances were unequal between groups, a Welch's ANOVA was used instead. If the data did not meet parametric assumptions, nonparametric tests were performed where applicable. For our second cohort, in some assays, genotype and cre were collapsed into a single factor with six levels for nonparametric tests. Though both sexes were included in our behavioral cohort, we were underpowered to include sex as a factor in our analyses. For repeated measures ANOVA, if sphericity was violated, Greenhouse–Geisser corrections were applied. Frequency analysis was performed using Fisher's exact test (since expected cell counts were not met). Post hoc analyses were performed using Bonferroni's, Dunnett T3's, or Dunn's depending on the respective parametric or nonparametric tests used. Comparisons for our first cohort were made among HET^Cre+^, HOM^Cre+^, and WT^Cre+^ (as controls). Based on results from our first cohort, largely planned comparisons were made between HOM^Cre+^ and HET^Cre+^, HET^Cre−^, WT^Cre+^, and WT^Cre−^. Appropriately adjusted *p* values were reported to correct for multiple comparisons. Significance was set at *p* < 0.05. Statistical analyses were performed using GraphPad Prism (Version 10.4.1) or IBM SPSS Statistics (Version 30). A detailed break‐down of statistical analyses and results with exact *p*‐values is available in Table S1.

## 3 Results

**FIGURE 1:**
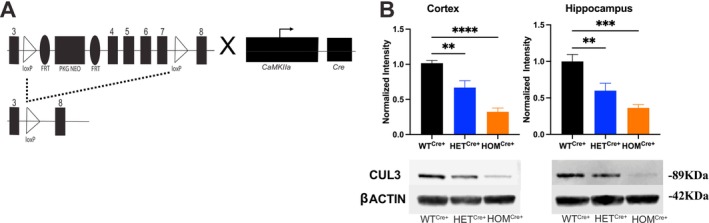
*Cul3* floxed mice and validation. (A) Schematic of *Cul3* floxed mice with loxP sites (along with frt and neo‐cassette) depicted after exon 3 and before exon 8. Crossing with mice expressing *CaMKIIa Cre‐recombinase* promoter excises exon 4–7 from the floxed allele in forebrain excitatory neurons, resulting in a frameshift. (B) Representative immunoblots with anti‐CUL3 antibody (anti‐β‐actin as loading control) from WT^Cre+^, HET^Cre+^, and HOM^Cre+^ cortical and hippocampal lysates showing decreased protein expression of CUL3. Reactive bands were identified at ~89 kDa for CUL3 and ~42 kDa for β‐actin.

We used the cre‐loxp system to generate a delayed, *Cul3* postnatal deletion from forebrain excitatory neurons. We crossed floxed *Cul3* mice with mice expressing Cre‐recombinase driven by a *CaMKIIa* promoter (Figure 1A); expression of *CaMKIIα* promoter‐driven Cre‐recombinase is reported to begin roughly around postnatal day 8, with expression increasing 10‐fold by the third week of postnatal development [29, 30]. Expression is predominantly localized to forebrain excitatory neurons of the cortex and hippocampi, and deletion likely occurs in the third or fourth week of postnatal development [29]. Using this strategy, we successfully generated viable conditional homozygous mice that survived into adulthood. This resulted in a significant loss of CUL3 protein expression in the cortex (Figure 1B: One‐way independent ANOVA: *F*_(2, 15)_ = 25.09, *p* < 0.0001; Bonferroni: WT^Cre+^ vs. HET^Cre+^, *p* < 0.01; WT^Cre+^ vs. HOM^Cre+^, *p* < 0.0001; HET^Cre+^ vs. HOM^Cre+^, *p* < 0.01) and hippocampus (Figure 1B: One‐way independent ANOVA: *F*_(2, 15)_ = 14.40, *p* < 0.001; Bonferroni: WT^Cre+^ vs. HET^Cre+^, *p* < 0.05; WT^Cre+^ vs. HOM^Cre+^, *p* < 0.001; HET^Cre+^ vs. HOM^Cre+^, *p* > 0.05), with homozygous, conditional *Cul3* KO mice showing an ~64%–68% decrease in CUL3 protein.

We examined multiple behavioral outcome measures across a broad range of behavioral domains, including those with supposed face validity for autism and its comorbidities. To increase confidence in our findings, behavioral assessments were performed on two independent cohorts. Our first cohort made use of all Cre+, *Cul3* wild‐type (WT), heterozygous (HET), and homozygous (HOM) conditional deletion littermates (WT^Cre+^, HET^Cre+^, and HOM^Cre+^). Most of these experiments were repeated in a second cohort that also included age‐matched Cre‐negative control groups (WT^Cre−^, HET^Cre−^, and HOM^Cre−^).

To ensure the overall well‐being of these mice, we measured weight at postnatal day 90 (P90) in our second cohort and found no changes across genotypes (Figure S1A: Two‐way independent ANOVA: Main effect of genotype, *F*_(2, 58)_ = 0.480, *p* > 0.05; Main effect of Cre, *F*_(1, 58)_ = 4.464, *p* < 0.05; Main effect of genotype × Cre, *F*_(2, 58)_ = 0.416, *p* > 0.05; Bonferroni: WT^Cre−^ vs. HOM^Cre+^, *p* > 0.05; HET^Cre−^ vs. HOM^Cre+^, *p* > 0.05; HOM^Cre−^ vs. HOM^Cre+^, *p* > 0.05; WT^Cre+^ vs. HOM^Cre+^, *p* > 0.05; HET^Cre+^ vs. HOM^Cre+^, *p* > 0.05). Table S1 notes exact *p* values and statistical results for these and all other experiments.

### 3.1 No Difference in Social Approach or Sociability Across Genotypes

**FIGURE 2:**
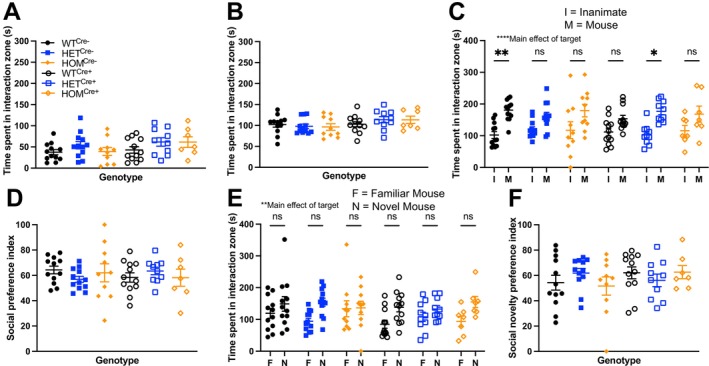
No change in social interaction in heterozygous or homozygous *Cul3* mice in two different social behaviors. (A) In a caged‐conspecific test, approach to an inanimate object was equivalent across groups in our second cohort. (B) No change in approach toward a social target across genotypes. (C) In a 3‐chamber test of sociability, an overall main effect of social target was observed across groups and (D) no change in social preference index. (E) An overall main effect of target was observed across all groups with post hoc tests between the familiar vs. novel mouse within a genotype showing no significant effect on preference for social novelty. (F) Social novelty preference index also demonstrated no change in discrimination among genotypes.

Because deficits in social interaction are a core feature of ASD, we sought to test social interaction in two different tasks, caged conspecific test (social approach) and social preference (a.k.a. sociability). In the social approach task for the first cohort, *Cul3* homozygotes showed decreased time spent interacting with an inanimate object in an open field (Figure S1B: One‐way independent ANOVA: Main effect of genotype, *F*_(2, 41)_ = 8.111, *p* < 0.01; Bonferroni: WT^Cre+^ vs. HET^Cre+^, *p* > 0.05; WT^Cre+^ vs. HOM^Cre+^, *p* < 0.05; HET^Cre+^ vs. HOM^Cre+^, *p* < 0.001). When a social target was introduced, however, the time spent in the interaction zone was similar across groups suggesting no change in social approach behavior (Figure S1C: One‐way independent ANOVA: Main effect of genotype, *F*_(2, 41)_ = 2.62, *p* > 0.05). When we repeated the social approach assay in our second cohort, we saw no changes in time spent interacting with the inanimate object (Figure 2A: Two‐way independent ANOVA: Main effect of genotype, *F*_(2, 57)_ = 2.267, *p* > 0.05; Main effect of cre, *F*_(1, 57)_ = 2.662, *p* = 0.05; Main effect of genotype × cre, *F*_(2, 57)_ = 0.493, *p* > 0.05) or the social target (Figure 2B: Two‐way independent ANOVA: Main effect of genotype, *F*_(2, 57)_ = 0.126; Main effect of cre, *F*_(1, 57)_ = 3.803, *p* > 0.05; Main effect of genotype × cre, *F*_(2, 57)_ = 0.936, *p* > 0.05). In essence, neither postnatal heterozygous nor homozygous *Cul3* deletion resulted in altered social approach behavior.

Next, we examined the effects of *Cul3* deletion in the 3‐chamber assay of “sociability” [31, 32]. In our first cohort during the habituation phase, we observed *Cul3* homozygotes showed a preference for one of the chambers unlike their littermate controls (Figure S1D: Two‐way RM ANOVA: Main effect of genotype, *F*_(2, 41)_ = 3.743, *p* < 0.05; Main effect of chamber, *F*_(1, 41)_ = 11.94, *p* < 0.01; Main effect of genotype × chamber, *F*_(2, 41)_ = 1.233, *p* > 0.05; Bonferroni: WT^Cre+^ Chamber A vs. Chamber B, *p* > 0.05; HET^Cre+^ Chamber A vs. Chamber B, *p* > 0.05; HOM^Cre+^ Chamber A vs. Chamber B, *p* < 0.05); all subsequent testing of social preferences were counterbalanced across target locations to avoid confounds. In the social versus inanimate (a.k.a. “sociability”) phase of the task, we observed that all Cre‐positive *Cul3* mice (WT/HET/HOM) showed a significant increase in preference for the social target compared to an inanimate target (Figure S1E: Two‐way RM ANOVA: Main effect of genotype, *F*_(2, 41)_ = 7.217, *p* < 0.01; Main effect of target, *F*_(1, 41)_ = 49.15, *p* < 0.0001; Main effect of genotype × target, *F*_(2, 41)_ = 6.174, *p* < 0.01; Bonferroni: WT^Cre+^ inanimate vs. mouse, *p* < 0.05; HET^Cre+^ inanimate vs. mouse, *p* < 0.05; HOM^Cre+^ inanimate vs. mouse, *p* < 0.0001). Intact sociability was also reflected by no changes in the social preference index (Figure S1F: One‐way independent ANOVA: Main effect of genotype *F*_(2, 41)_ = 4.487, *p* < 0.05; Bonferroni: WT^Cre+^ vs. HET^Cre+^, *p* > 0.05; WT^Cre+^ vs. HOM^Cre+^, *p* < 0.05; HET^Cre+^ vs. HOM^Cre+^, *p* < 0.05). In the final phase of the assay, testing preference for social novelty and social recognition memory, a novel social target was introduced in the other chamber and preference for the familiar versus the novel social target was recorded. We did not observe a preference for the novel target across all groups (Figure S1G: Two‐way RM ANOVA: Main effect of genotype, *F*_(2, 41)_ = 1.782, *p* > 0.05; Main effect of target, *F*_(1, 41)_ = 1.572, *p* > 0.05; Main effect of genotype × chamber, *F*_(2, 41)_ = 0.07413, *p* > 0.05). This was also reflected in social novelty discrimination index (Figure S1H; One‐way independent ANOVA: Main effect of genotype, *F*_(2, 41)_ = 0.2182, *p* > 0.05). While these data indicated no change in social approach or sociability, we also examined basic olfaction. We observed a slight, significant increase in the latency to find food in *Cul3* homozygotes compared to their littermate counterparts (Figure S1I: One‐way independent ANOVA: Main effect of genotype, *F*_(2, 41)_ = 4.838, *p* < 0.05; Bonferroni: WT^Cre+^ vs. HET^Cre+^, *p* > 0.05; WT^Cre+^ vs. HOM^Cre+^, *p* < 0.05; HET^Cre+^ vs. HOM^Cre+^, *p* < 0.05).

To confirm the lack of changes in sociability, we repeated this task in our second cohort. In the 3‐chamber social interaction habituation phase, we observed no change in preference for the empty chambers (Figure S1J: Three‐way RM ANOVA: Main effect of genotype, *F*_(2, 57)_ = 0.728, *p* > 0.05; Main effect of cre, *F*_(1, 57)_ = 0.049, *p* > 0.05; Main effect of genotype × cre, *F*_(2, 57)_ = 0.148, *p* > 0.05; Main effect of chamber, *F*_(1, 57)_ = 1.546, *p* > 0.05; Main effect of chamber × genotype, *F*_(2, 57)_ = 0.373; Main effect of chamber × cre, *F*_(1, 57)_ = 0.062, *p* > 0.05; Main effect of chamber × cre × genotype, *F*_(2, 57)_ = 1.730, *p* > 0.05). As before, no differences were observed in the test of sociability as there was an overall main effect of social target (Figure 2C: Three‐way RM ANOVA: Main effect of genotype, *F*_(2, 57)_ = 0.719, *p* > 0.05; Main effect of cre, *F*_(1, 57)_ = 1.054, *p* > 0.05; Main effect of genotype × cre, *F*_(2, 57)_ = 0.193, *p* > 0.05; Main effect of target, *F*_(1, 57)_ = 30.344, *p* < 0.0001; Main effect of target × genotype, *F*_(2, 57)_ = 0.008, *p* > 0.05; Main effect of target × cre, *F*_(1, 57)_ = 0.043, *p* > 0.05; Main effect of target × cre × genotype, *F*_(2, 57)_ = 1.035, *p* > 0.05). Similarly, no changes were observed in the social preference index (Figure 2D: Two‐way independent ANOVA: Main effect of genotype, *F*_(2, 57)_ = 0.052, *p* > 0.05; Main effect of cre, *F*_(1, 57)_ = 0.089, *p* > 0.05; Main effect of genotype × cre, *F*_(2, 57)_ = 1.287, *p* > 0.05). In the preference for social novelty and social recognition phase, we found a significance of preference for the novel social target (Figure 2E: Three‐way RM ANOVA: Main effect of genotype, *F*_(2, 57)_ = 0.484, *p* > 0.05; Main effect of cre, *F*_(1, 57)_ = 3.732, *p* > 0.05; Main effect of genotype × cre, *F*_(2, 57)_ = 0.827, *p* > 0.05; Main effect of target, *F*_(1, 57)_ = 9.850, *p* < 0.001; Main effect of target × genotype, *F*_(2, 57)_ = 0.062, *p* > 0.05; Main effect of target × cre, *F*_(1, 57)_ = 0.392, *p* > 0.05; Main effect of target × cre × genotype, *F*_(2, 57)_ = 1.207, *p* > 0.05) indicating intact preference for social novelty. The social novelty preference index confirmed no change in preference for social novelty among groups (Figure 2F: Two‐way independent ANOVA: Main effect of genotype, *F*_(2, 57)_ = 0.054, *p* > 0.05; Main effect of cre, *F*_(1, 57)_ = 0.944, *p* > 0.05; Main effect of genotype × cre, *F*_(2, 57)_ = 1.319, *p* > 0.05). Our data suggest that delayed *Cul3* deletion in forebrain excitatory neurons does not affect sociability or social novelty preference.

### 3.2 Increased Repetitive Jumping and Decreased Marble Burying

**FIGURE 3:**
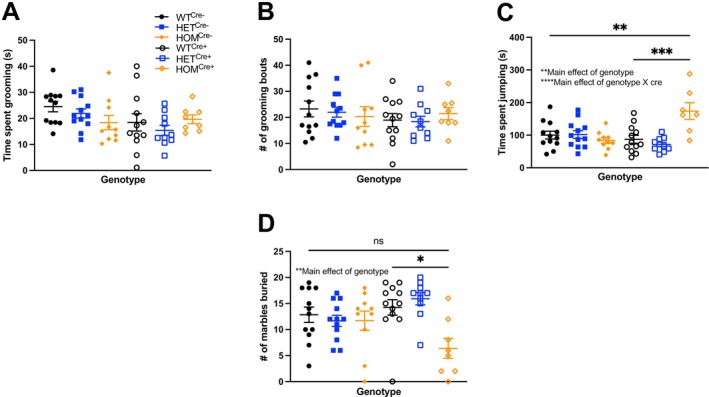
Repetitive behavior assays (grooming, repetitive jumping, and marble burying). (A) Time spent grooming and (B) number of grooming bouts is similar across genotypes. (C) Significant increase in repetitive jumping observed in *Cul3* homozygous conditional knockouts versus WT controls. (D) Decreased marble burying was observed in HOM^Cre+^.

Because repetitive behavior is another core feature of ASD [33, 34], we examined time spent self‐grooming, an ethologically relevant, repeated behavior in mice. Using the time spent grooming (Figure S2A: One‐way independent ANOVA: Main effect of genotype, *F*_(2, 41)_ = 0.115, *p* > 0.05) and number of grooming bouts (Figure S2B: Main effect of genotype: *F*_(2, 41)_ = 1.541, *p* > 0.05), we found no change in grooming behavior in our initial cohort among all genotypes. When this experiment was repeated with Cre‐negative controls in our second cohort, we also observed no change in grooming measures (Figure 3A: Two‐way independent ANOVA: Main effect of genotype, *F*_(2, 58)_ = 0.902, *p* > 0.05; Main effect of cre, *F*_(1, 58)_ = 3.566, *p* > 0.05; Main effect of genotype × cre, *F*_(2, 58)_ = 1.486, *p* > 0.05; Figure 3B: Two‐way independent ANOVA: Main effect of genotype, *F*_(2, 58)_ = 0.065, *p* > 0.05; Main effect of cre, *F*_(1, 58)_ = 1.045, *p* > 0.05; Main effect of genotype × cre, *F*_(2, 58)_ = 0.539, *p* > 0.05).

An observation in our initial cohort suggested an increase in repetitive jumping behavior in *Cul3* homozygotes. To quantify this behavior in a rigorous manner, we scored for repetitive jumping during a nest‐building assay for a 30‐min period. We observed an increase in repetitive jumping by Cre‐positive *Cul3* homozygotes (Figure 3C: Two‐way independent ANOVA: Main effect of genotype, *F*_(2, 57)_ = 5.584, *p* < 0.001; Main effect of cre, *F*_(1, 57)_ = 2.227, *p* > 0.05; Main effect of genotype × cre, *F*_(2, 57)_ = 11.544, *p* < 0.0001; Bonferroni: WT^Cre−^ vs. HOM^Cre+^, *p* < 0.01; HET^Cre−^ vs. HOM^Cre+^, *p* < 0.01; HOM^Cre−^ vs. HOM^Cre+^, *p* < 0.001; WT^Cre+^ vs. HOM^Cre+^, *p* < 0.001; HET^Cre+^ vs. HOM^Cre+^, *p* < 0.0001) compared to their age‐matched controls.

In the marble burying assay, a test often interpreted as either repetitive behavior [35] or anxiety‐related behavior [36], *Cul3* homozygotes showed a decrease in the number of marbles that were buried (Figure S2C: Independent samples Kruskal–Wallis test: Main effect of genotype, *H*_(2)_ = 19.588, *p* < 0.0001; Dunn's: WT^Cre+^ vs. HET^Cre+^, *p* > 0.05; WT^Cre+^ vs. HOM^Cre+^, *p* < 0.001; HET^Cre+^ vs. HOM^Cre+^, *p* < 0.001). We repeated this experiment with Cre‐negative controls and again found a main effect of genotype (Figure 3D: Independent samples Kruskal–Wallis test: Main effect of genotype, *H*_(5)_ = 16.205, *p* < 0.01; Dunn's: WT^Cre−^ vs. HOM^Cre+^, *p* > 0.05; HET^Cre−^ vs. HOM^Cre+^, *p* > 0.05; HOM^Cre−^ vs. HOM^Cre+^, *p* > 0.05; WT^Cre+^ vs. HOM^Cre+^, *p* < 0.05; HET^Cre+^ vs. HOM^Cre+^, *p* < 0.01) confirming decreased marble burying.

### 3.3 Increased Locomotor Activity

**FIGURE 4:**
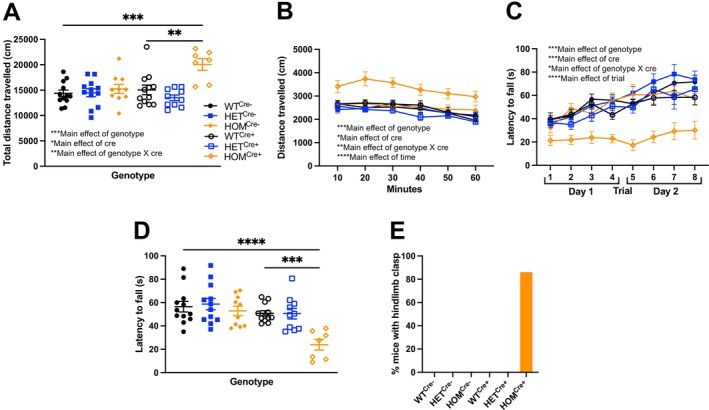
Increased locomotor activity, hindlimb clasping, and impaired motor coordination in Cre‐positive *Cul3* homozygotes. (A) Cumulative distance traveled is significantly increased in *Cul3* homozygous deletion mice compared to age‐matched controls. (B) Distance traveled in 10‐min bins shows increased locomotor activity in Cre‐positive *Cul3* homozygotes. (C) Accelerating rotarod task demonstrates latency to fall across 8 trials is significantly decreased in Cre‐positive *Cul3* homozygotes. (D) Average latency to fall per mouse is plotted across groups. (E) 85% of *Cul3* homozygous mice show hindlimb clasping compared to age‐matched controls.

In our initial cohort, HOM^Cre+^ mice demonstrated a strong trend toward increased locomotor activity in an open field compared to their littermate controls (Figure S2D: Welch's ANOVA: Main effect of genotype, *W*_(2, 15.83)_ = 3.456, *p* = 0.0568). As a result, in our second cohort we increased time spent in the open field from 20 to 60 min. Total cumulative distance traveled was significantly increased in *Cul3* homozygotes compared to all other groups (Figure 4A: Two‐way independent ANOVA: Main effect of genotype, *F*_(2, 57)_ = 9.502, *p* < 0.001; Main effect of cre, *F*_(1, 57)_ = 4.745, *p* < 0.05; Main effect of genotype × cre, *F*_(2, 57)_ = 5.644, *p* < 0.01; Bonferroni: WT^Cre−^ vs. HOM^Cre+^, *p* < 0.001; HET^Cre−^ vs. HOM^Cre+^, *p* < 0.001; HOM^Cre−^ vs. HOM^Cre+^, *p* < 0.01; WT^Cre+^ vs. HOM^Cre+^, *p* < 0.01; HET^Cre+^ vs. HOM^Cre+^, *p* < 0.0001). This was still the case when locomotor activity was plotted in 10‐min bins (Figure 4B: Three‐way RM ANOVA: Main effect of genotype, *F*_(2, 57)_ = 9.502, *p* < 0.001; Main effect of cre, *F*_(1, 57)_ = 4.745, *p* < 0.05; Main effect of genotype × cre, *F*_(2, 57)_ = 5.644, *p* < 0.01; Main effect of time, *F*_(3.253, 185.420)_ = 27.978, *p* < 0.0001; Main effect of time × genotype, *F*_(6.506, 185.420)_ = 1.087, *p* > 0.05; Main effect of time × cre, *F*_(3.253, 185.420)_ = 1.570, *p* > 0.05; Main effect of time × genotype × cre, *F*_(6.506, 185.420)_ = 1.692, *p* > 0.05).

### 3.4 Decreased Motor Coordination

Motor coordination or motor learning differences are sometimes comorbid with ASD [37]. In a cohort of human patients with pathological variants of *CUL3* mutations, 86% exhibited motor delays [8]. We tested motor learning and coordination on the accelerating rotarod task. In our initial cohort, *Cul3* homozygotes showed significantly decreased motor coordination and learning compared to their littermate controls across eight trials (Figure S3A: Two‐way RM ANOVA: Main effect of genotype, *F*_(2, 41)_ = 15.35, *p* < 0.0001; Main effect of trials, *F*_(5.053, 207.2)_ = 4.055, *p* < 0.01; Main effect of genotype × trials, *F*_(14, 287)_ = 2.036, *p* < 0.05). This was consistent when the latency to fall was plotted as total latency to fall per mouse across all trials (Figure S3B: One‐way independent ANOVA: Main effect of genotype, *F*_(2, 41)_ = 15.35, *p* < 0.0001; Bonferroni: WT^Cre+^ vs. HET^Cre+^, *p* < 0.001; WT^Cre+^ vs. HOM^Cre+^, *p* < 0.00001; HET^Cre+^ vs. HOM^Cre+^, *p* > 0.05). In the second cohort, we initiated the accelerating rotarod task at 0 rpm increasing to 45 rpm (instead of starting at 4 rpm as with the first cohort). We again observed motor coordination impairments in HOM^Cre+^ mice compared to controls across trials (Figure 4C: Three‐way RM ANOVA: Main effect of genotype, *F*_(2, 57)_ = 7.910, *p* < 0.001; Main effect of cre, *F*_(1, 57)_ = 16.244, *p* < 0.001; Main effect of genotype × cre, *F*_(2, 57)_ = 3.949, *p* < 0.05; Main effect of trial, *F*_(5.022, 286.263)_ = 22.527, *p* < 0.0001; Main effect of trial × genotype, *F*_(10.044, 286.263)_ = 1.631, *p* > 0.05; Main effect of trial × cre, *F*_(5.022, 286.263)_ = 2.034, *p* > 0.05; Main effect of trial × genotype × cre, *F*_(10.044, 286.263)_ = 0.776, *p* > 0.05), where Cre‐positive *Cul3* homozygotes, again, showed significantly lower average latency to fall (Figure 4D: Two‐way independent ANOVA: Main effect of genotype, *F*_(2, 57)_ = 7.910, *p* < 0.001; Main effect of cre, *F*_(1, 57)_ = 16.244, *p* < 0.001; Main effect of genotype × cre, *F*_(2, 57)_ = 3.949, *p* < 0.05; Bonferroni: WT^Cre−^ vs. HOM^Cre+^, *p* < 0.0001; HET^Cre−^ vs. HOM^Cre+^, *p* < 0.0001; HOM^Cre−^ vs. HOM^Cre+^, *p* < 0.001; WT^Cre+^ vs. HOM^Cre+^, *p* < 0.001; HET^Cre+^ vs. HOM^Cre+^, *p* < 0.01).

Based on our initial findings, we sought to understand if motor coordination impairments were robust and could be replicated in a separate task—the foot misplacement assay. *Cul3* homozygotes showed no change in the number of forelimb slips (Figure S3C: Independent samples Kruskal–Wallis test: Main effect of genotype, *H*_(5)_ = 9.358, *p* > 0.05) and hindlimb slips when compared to controls (Figure S3D: Independent samples Kruskal–Wallis test: Main effect of genotype, *H*_(5)_ = 2.835, *p* > 0.05). Latency to cross the ladder was also similar across groups (Figure S3E: Independent samples Kruskal–Wallis test: Main effect of genotype, *H*_(5)_ = 6.007, *p* > 0.05). Thus, motor coordination was impaired on the accelerating rotarod task but not on the somewhat easier task of horizontal ladder walking.

We also investigated if grip strength could be a potential confounding factor in the rotarod task as latency to fall could be influenced by the animal's ability to grip and stay on the rod. Grip strength remained intact across all groups suggesting it did not affect performance on the rotarod (Figure S3F: Two‐way independent ANOVA: Main effect of genotype, *F*_(2, 58)_ = 0.531, *p* > 0.05; Main effect of cre, *F*_(1, 58)_ = 5.274, *p* < 0.05; Main effect of genotype × cre, *F*_(2, 58)_ = 0.428, *p* > 0.05).

In our initial cohort, we noticed that 100% of *Cul3* homozygous deletion mice showed increased hindlimb clasping when picked up by their tail compared to WT littermate controls, a statistically significant difference (Figure S3G,H: Fisher's exact test, *p* < 0.0001). Repeating this in our second cohort, we observed that 85% of Cre‐positive homozygous mutants showed hindlimb clasping, a significant increase compared to both WT and heterozygous controls (Figure 4E: Fisher's exact test, *p* < 0.0001).

### 3.5 Learning and Memory

ASD is often comorbid with ID [38]. In a recent study, 77% of patients who displayed CUL3‐related NDD were diagnosed with ID [8]. To assess learning and memory impairments, in our first cohort, we subjected mice to fear conditioning and tested them on context and cue‐dependent fear memory. In the hippocampus‐dependent contextual fear memory task, *Cul3* homozygotes showed decreased freezing compared to heterozygotes and WTs, suggesting impaired contextual fear memory (Figure S4A: One‐way independent ANOVA: Main effect of genotype, *F*_(2, 41)_ = 9.097, *p* < 0.001; Bonferroni: WT^Cre+^ vs. HET^Cre+^, *p* > 0.05; WT^Cre+^ vs. HOM^Cre+^, *p* < 0.01; HET^Cre+^ vs. HOM^Cre+^, *p* < 0.001). Cued‐fear memory, however, remained intact across all groups (Figure S4B: One‐way independent ANOVA: Main effect of genotype, *F*_(2, 41)_ = 0.2997, *p* > 0.05). We did not repeat this experiment in our second cohort.

In addition, we tested mice on another hippocampus‐dependent spatial learning and memory assay, the Morris water maze. *Cul3* homozygotes showed significant impairment in spatial learning compared to littermate controls in the latency to reach the hidden platform across 11 days (Figure S4C: Two‐way RM ANOVA: Main effect of genotype, *F*_(2, 37)_ = 58.995, *p* < 0.0001; Main effect of day, *F*_(2, 228.680)_ = 32.683, *p* < 0.0001; Main effect of genotype × day, *F*_(12.361, 228.680)_ = 2.90, *p* < 0.0001), where *Cul3* homozygotes showed increased average latency to platform (Figure S4D: Welch's ANOVA: Main effect of genotype *W*_(2, 11.82)_ = 15.54, *p* < 0.001; Dunnett T3: WT^Cre+^ vs. HET^Cre+^, *p* > 0.05; WT^Cre+^ vs. HOM^Cre+^, *p* < 0.01; HET^Cre+^ vs. HOM^Cre+^, *p* < 0.01). This corroborated with distance traveled before reaching the hidden platform during training, which was increased in *Cul3* homozygotes (Figure S4E: Two‐way RM ANOVA: Main effect of genotype, *F*_(2, 37)_ = 22.015, *p* < 0.0001; Main effect of day, *F*_(6.295, 232.930)_ = 18.037, *p* < 0.0001; Main effect of genotype × day, *F*_(12.591, 232.930)_ = 3.594, *p* < 0.0001), while the overall average distance traveled was significantly increased in *Cul3* homozygotes compared to littermate controls (Figure S4F: One‐way independent ANOVA: Main effect of genotype, *F*_(2, 37)_ = 22.015, *p* < 0.0001; Bonferroni: WT^Cre+^ vs. HET^Cre+^, *p* > 0.05; WT^Cre+^ vs. HOM^Cre+^, *p* < 0.0001; HET^Cre+^ vs. HOM^Cre+^, *p* < 0.0001). Though *Cul3* homozygotes had significantly lower swim speed compared to controls across training days (Figure S4G: Two‐way RM ANOVA: Main effect of genotype, *F*_(2, 37)_ = 8.955, *p* < 0.001; Main effect of day, *F*_(5.393, 199.537)_ = 1.246, *p* > 0.05; Main effect of day × genotype, *F*_(10.786, 199.537)_ = 3.247, *p* < 0.0001) and average swim speed also being significantly decreased (Figure S4H: One‐way independent ANOVA: Main effect of genotype, *F*_(2, 37)_ = 8.955, *p* < 0.001; Bonferroni: WT^Cre+^ vs. HET^Cre+^, *p* > 0.05; WT^Cre+^ vs. HOM^Cre+^, *p* < 0.001; HET^Cre+^ vs. HOM^Cre+^, *p* < 0.01), the learning deficit remained when we eliminated swim speed as a confounding factor using distance traveled as the measure of learning.

In the probe trial, spatial memory was impaired in the *Cul3* homozygous mutants; time spent in the target quadrant was significantly lower compared to littermate controls (Figure S4I: Two‐way RM ANOVA: Main effect of genotype, *F*_(2, 37)_ = 0.404, *p* > 0.05; Main effect of quadrant, *F*_(2.363, 87.428)_ = 22.154, *p* < 0.0001; Main effect of genotype × quadrant, *F*_(4.726, 87.428)_ = 4.680, *p* < 0.001; Bonferroni: Target quadrant, WT^Cre+^ vs. HET^Cre+^, *p* > 0.05; WT^Cre+^ vs. HOM^Cre+^, *p* < 0.001; HET^Cre+^ vs. HOM^Cre+^, *p* < 0.01; Opposite quadrant, WT^Cre+^ vs. HET^Cre+^, *p* > 0.05; WT^Cre+^ vs. HOM^Cre+^, *p* < 0.01; HET^Cre+^ vs. HOM^Cre+^, *p* < 0.01; Right adjacent, WT^Cre+^ vs. HET^Cre+^, *p* > 0.05; WT^Cre+^ vs. HOM^Cre+^, *p* > 0.05; HET^Cre+^ vs. HOM^Cre+^, *p* > 0.05; Left adjacent, WT^Cre+^ vs. HET^Cre+^, *p* > 0.05; WT^Cre+^ vs. HOM^Cre+^, *p* > 0.05; HET^Cre+^ vs. HOM^Cre+^, *p* > 0.05). These results suggest mice were slower to learn a spatial task and were less adept at using a spatial strategy to recall a spatial memory 24 h after training.

**FIGURE 5:**
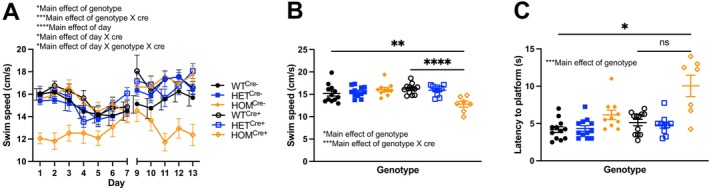
Decreased swim speed and increased latency to visible platform in the Morris water maze. (A) Swim speed was significantly decreased in Cre‐positive *Cul3* homozygotes across 12 days of training. (B) Average swim speed across all trials for each mouse showed significantly decreased swim speed in *Cul3* homozygotes. (C) Latency to visible platform was significantly increased in HOM^Cre+^ compared to WT controls.

We repeated the Morris water maze in our second cohort to test if our findings were robust. We again observed that Cre‐positive *Cul3* homozygotes showed an increase in latency to the hidden platform compared to age‐matched controls across 12 days of training (Figure S5A: Three‐way RM ANOVA: Main effect of genotype, *F*_(2, 57)_ = 18.972, *p* < 0.0001; Main effect of cre, *F*_(1, 57)_ = 8.296, *p* < 0.01; Main effect of genotype × cre, *F*_(2, 57)_ = 10.837, *p* < 0.001; Main effect of day, *F*_(8.209, 467.908)_ = 23.400, *p* < 0.0001; Main effect of day × genotype, *F*_(16.418, 467.908)_ = 1.151, *p* > 0.05; Main effect of day × cre, *F*_(8.209, 467.908)_ = 0.596, *p* > 0.05; Main effect of day × genotype × cre, *F*_(16.418, 467.908)_ = 0.672, *p* > 0.05) where average latency to platform was also increased in Cre‐positive *Cul3* homozygotes (Figure S5B: Two‐way independent ANOVA: Main effect of genotype, *F*_(2, 57)_ = 18.972, *p* < 0.0001; Main effect of cre, *F*_(1, 57)_ = 8.296, *p* < 0.01; Main effect of genotype × cre, *F*_(2, 57)_ = 10.837, *p* < 0.001; Bonferroni: WT^Cre−^ vs. HOM^Cre+^, *p* < 0.0001; HET^Cre−^ vs. HOM^Cre+^, *p* < 0.0001; HOM^Cre−^ vs. HOM^Cre+^, *p* < 0.0001; WT^Cre+^ vs. HOM^Cre+^, *p* < 0.0001; HET^Cre+^ vs. HOM^Cre+^, *p* < 0.0001). An overall small significant main effect of genotype was observed in the distance traveled (Figure S5C: Three‐way RM ANOVA: Main effect of genotype, *F*_(2, 57)_ = 3.538, *p* < 0.05; Main effect of cre, *F*_(1, 57)_ = 1.204, *p* > 0.05; Main effect of genotype × cre, *F*_(2, 57)_ = 0.736, *p* > 0.05; Main effect of day, *F*_(7.957, 453.568)_ = 13.392, *p* < 0.0001; Main effect of day × genotype, *F*_(15.915, 453.568)_ = 1.239, *p* > 0.05; Main effect of day × cre, *F*_(7.957, 453.568)_ = 1.078, *p* > 0.05; Main effect of day × genotype × cre, *F*_(15.915, 453.568)_ = 0.672, *p* > 0.05), where average distance traveled showed a trend toward increase in HOM^Cre+^ compared with WT^Cre+^ and WT^Cre−^ controls (Figure S5D: Two‐way independent ANOVA: Main effect of genotype, *F*_(2, 57)_ = 3.538, *p* < 0.05; Main effect of cre, *F*_(1, 57)_ = 1.204, *p* > 0.05; Main effect of genotype × cre, *F*_(2, 57)_ = 0.736, *p* > 0.05; Bonferroni: WT^Cre−^ vs. HOM^Cre+^, *p* = 0.08; HET^Cre−^ vs. HOM^Cre+^, *p* > 0.05; HOM^Cre−^ vs. HOM^Cre+^, *p* > 0.05; WT^Cre+^ vs. HOM^Cre+^, *p* = 0.08; HET^Cre+^ vs. HOM^Cre+^, *p* > 0.05). Swim speed was significantly decreased in Cre‐positive homozygous mice compared to controls across training days (Figure 5A: Three‐way RM ANOVA: Main effect of genotype, *F*_(2, 57)_ = 4.594, *p* < 0.05; Main effect of cre, *F*_(1, 57)_ = 2.797, *p* > 0.05; Main effect of genotype × cre, *F*_(2, 57)_ = 11.385, *p* < 0.001; Main effect of day, *F*_(7.311, 416.744)_ = 12.210, *p* < 0.0001; Main effect of day × genotype, *F*_(14.623, 416.744)_ = 1.319, *p* > 0.05; Main effect of day × cre, *F*_(7.311, 416.744)_ = 2.303, *p* < 0.05; Main effect of day × genotype × cre, *F*_(14.623, 416.744)_ = 1.974, *p* < 0.05), where average swim speed was significantly decreased in HOM^Cre+^ compared to WT controls, again a robust and reproducible finding (Figure 5B: Two‐way independent ANOVA: Main effect of genotype, *F*_(2, 57)_ = 4.594, *p* < 0.05; Main effect of cre, *F*_(1, 57)_ = 2.797, *p* > 0.05; Main effect of genotype × cre, *F*_(2, 57)_ = 11.385, *p* < 0.0001; Bonferroni: WT^Cre−^ vs. HOM^Cre+^, *p* < 0.01; HET^Cre−^ vs. HOM^Cre+^, *p* < 0.01; HOM^Cre−^ vs. HOM^Cre+^, *p* < 0.001; WT^Cre+^ vs. HOM^Cre+^, *p* < 0.0001; HET^Cre+^ vs. HOM^Cre+^, *p* < 0.001).

Although spatial learning was significantly decreased in the Morris water maze task in HOM^Cre+^ mice, in the initial probe trial on Day 8 (Figure S5E: Three‐way RM ANOVA: Main effect of genotype, *F*_(2, 57)_ = 2.081, *p* > 0.05; Main effect of cre, *F*_(1, 57)_ = 0.204, *p* > 0.05; Main effect of genotype × cre, *F*_(2, 57)_ = 1.621, *p* > 0.05; Main effect of quadrant, *F*_(2.485, 141.641)_ = 14.782, *p* < 0.0001; Main effect of quadrant × genotype, *F*_(4.970, 141.641)_ = 0.902, *p* > 0.05; Main effect of quadrant × cre, *F*_(2.485, 141.641)_ = 0.533, *p* > 0.05; Main effect of quadrant × genotype × cre, *F*_(4.970, 141.641)_ = 1.741, *p* > 0.05) we found an overall main effect of quadrant and no main effect of genotype suggesting no difference between genotypes in the time spent in the target quadrant. This was also the case on a second probe trial on Day 14 (Figure S5F: Three‐way RM ANOVA: Main effect of genotype, *F*_(2, 57)_ = 0.173, *p* > 0.05; Main effect of cre, *F*_(1, 57)_ = 0.997, *p* > 0.05; Main effect of genotype × cre, *F*_(2, 57)_ = 1.323, *p* > 0.05; Main effect of quadrant, *F*_(2.485, 141.641)_ = 14.782, *p* < 0.0001; Main effect of quadrant × genotype, *F*_(4.970, 141.641)_ = 0.902, *p* < 0.05; Main effect of quadrant × cre, *F*_(2.485, 141.641)_ = 0.533, *p* < 0.05; Main effect of quadrant × genotype × cre, *F*_(4.970, 141.641)_ = 1.741, p < 0.05). Overall, results of the Morris water maze test demonstrated consistently decreased spatial learning while the ability to use a spatial strategy in the probe trial was inconsistent across the two cohorts.

To eliminate the possibility of visual acuity affecting performance on the Morris water maze, mice were tested on the visible platform version of the water maze. In our first cohort, latency to reach the visible platform showed a trend toward a significant increase in homozygous mutants (Figure S5G: Independent samples Kruskal–Wallis test: Main effect of genotype, *H*_(2)_ = 5.247, *p* = 0.07). In our second cohort, we found that Cre‐positive *Cul3* homozygotes showed significantly higher latency to the platform compared with other groups, suggesting visual acuity could be a potential confound (Figure 5C: Welch's ANOVA: Main effect of genotype, *W*_(5.000, 24.04)_ = 4.042, *p* < 0.01).

Overall, our results on the Morris water maze across both cohorts demonstrate a decreased learning rate; the probe trial findings were inconsistent; swim speed and visual acuity were noted as confounds.

### 3.6 No Change in Anxiety‐Like Behaviors

In the human population, ASD can be comorbid with anxiety [39, 40]. In other rodent models of *Cul3*‐deficient mice, anxiety‐like behaviors showed mixed results [16, 18]. We tested mice on multiple tests that measure anxiety‐like behaviors.

On the elevated zero maze, *Cul3* homozygotes spent significantly more time in the open arms compared to heterozygotes and WT controls (Figure S6A: One‐way independent ANOVA: Main effect of genotype, *F*_(2, 41)_ = 19.68, *p* < 0.0001; Bonferroni: WT^Cre+^ vs. HET^Cre+^, *p* > 0.05; WT^Cre+^ vs. HOM^Cre+^, *p* < 0.0001; HET^Cre+^ vs. HOM^Cre+^, *p* < 0.0001). In our second cohort, we were unable to replicate this effect and found Cre‐positive *Cul3* homozygotes spent equivalent time compared to controls (Figure S6B: Two‐way independent ANOVA: Main effect of genotype, *F*_(2, 58)_ = 1.310, *p* > 0.05; Main effect of cre, *F*_(1, 58)_ = 0.174, *p* > 0.05; Main effect of genotype × cre, *F*_(2, 58)_ = 3.863, *p* < 0.05; Bonferroni: WT^Cre−^ vs. HOM^Cre+^, *p* > 0.05; HET^Cre−^ vs. HOM^Cre+^, *p* > 0.05; HOM^Cre−^ vs. HOM^Cre+^, *p* > 0.05; WT^Cre+^ vs. HOM^Cre+^, *p* > 0.05; HET^Cre+^ vs. HOM^Cre+^, *p* > 0.05).

In the open field task, *Cul3* homozygotes spent significantly more time along the edges (thigmotaxis) compared to their littermate controls which is often interpreted as an increase in anxiety‐like behavior (Figure S6C: Independent samples Kruskal–Wallis test: Main effect of genotype: *H*_(2)_ = 7.321, *p* < 0.05; Dunn's: WT^Cre+^ vs. HET^Cre+^, *p* > 0.05; WT^Cre+^ vs. HOM^Cre+^, *p* < 0.05; HET^Cre+^ vs. HOM^Cre+^, *p* > 0.05). Again, when we repeated this task on our second cohort, we were unable to replicate our findings from our initial cohort (Figure S6D: Two‐way independent ANOVA: Main effect of genotype, *F*_(2, 58)_ = 1.046, *p* > 0.05; Main effect of cre, *F*_(1, 58)_ = 0.067, *p* > 0.05; Main effect of genotype × cre, *F*_(2, 58)_ = 0.278, *p* > 0.05).

In the dark–light box, latency to enter the light chamber (Figure S6E: Independent samples Kruskal–Wallis test: Main effect of genotype, *H*_(2)_ = 3.573, *p* > 0.05) or time spent in the light chamber (Figure S6F: One‐way independent ANOVA: Main effect of genotype, *F*_(2, 41)_ = 2.130, *p* > 0.05) was unchanged across groups in our first cohort. In our second cohort, we again observed no overall change in the latency to enter the light chamber (Figure S6G: Independent samples Kruskal–Wallis test: Main effect of genotype, *H*_(5)_ = 0.879, *p* > 0.05) or time spent in the light chamber across all genotypes (Figure S6H: Two‐way independent ANOVA: Main effect of genotype, *F*_(2, 58)_ = 1.381, *p* > 0.05; Main effect of cre, *F*_(1, 58)_ = 0.476, *p* > 0.05; Main effect of genotype × cre, *F*_(2, 58)_ = 1.685, *p* > 0.05). Because we saw inconsistent and largely negative results, we conclude no change in anxiety‐like behavior in Cre‐positive *Cul3* homozygotes.

### 3.7 No Change in Nestbuilding

Nestbuilding is an ethologically relevant behavior [41, 42]. In some rodent models of autism [26, 27], the ability to build nests is impaired. On the nest building assay, in our first cohort, 24 h after a nestlet was introduced, *Cul3* homozygotes showed decreased nest height (Figure S7A: One‐way independent ANOVA: Main effect of genotype, *F*_(2, 41)_ = 12.49, *p* < 0.0001) compared to their littermate controls. However, the width of the nestlet (Figure S7B: Main effect of genotype, *F*_(2, 41)_ = 0.13148, *p* > 0.05) was similar across groups, possibly suggesting impaired nest building. However, in our second cohort, nestbuilding remained intact—the nests were of comparable height across groups (Figure S7C: Independent samples Kruskal–Wallis test: Main effect of genotype, *H*_(5)_ = 2.545, *p* > 0.05) and width (Figure S7D: Two‐way independent ANOVA: Main effect of genotype, *F*_(2, 57)_ = 0.009, *p* > 0.05; Main effect of cre, *F*_(1, 57)_ = 0.040, *p* > 0.05; Main effect of genotype × cre, *F*_(2, 57)_ = 1.33, *p* > 0.05).

## 4 Discussion

Ours is the first study to demonstrate effects on multiple behavioral outcome measures in a homozygous, forebrain‐specific, postnatal deletion of *Cul3*. We examined behaviors in two different cohorts to ensure robust and replicable behavioral outcomes. Although behaviors with potential face validity for ASD did not show clear changes (social interaction, repetitive behaviors), we found differences in multiple behavioral measures that were internally replicated across two cohorts with appropriate controls. Overall, our results demonstrate that postnatal homozygous *Cul3* deletion from forebrain excitatory neurons shows robust motor coordination impairments, repetitive jumping, increased locomotor activity, and decreased marble burying. These behavioral findings set the stage for future studies aimed at delineating mechanisms underlying *Cul3*‐deletion's effects on brain function and their potential relevance to behavioral outcomes.

Our study showed no change in overall body weight of Cre‐positive *Cul3* homozygotes in early adulthood (P90). Interestingly, other cell‐type specific early embryonic deletion of *Cul3* rodent models shows a decrease in body weight, brain size, and cortical thickness [12, 13, 18, 19]. Future studies are warranted, however, to understand the effects of postnatal homozygous *Cul3* deletion on brain size and cortical thickness.

In assays that test anxiety‐like behavior, we saw mixed results in our first cohort and no change across three different assays in our second cohort. We interpret that as no change in anxiety‐like measures. On another assay, nest building, we again observed inconsistent results between the first and second cohort. In our initial cohort, Cre‐positive *Cul3* homozygotes showed decreased height in the nests that were built but no change in the width. In our second cohort, however, no change in the height or width of the nest was observed across groups, demonstrating no consistent effect on nest building behavior.

Our data show no overall change in social interaction measures in two different assays. In the caged conspecific assay, a measure of social approach, there was no change in overall time spent in the interaction zone across both cohorts when a social target was introduced in an open field. In our initial cohort, *Cul3* homozygous deletion mice demonstrated an initial decrease in time spent near the inanimate target (empty cage); this was not observed in the second cohort. In the 3‐chamber assay of so‐called sociability, we observed a preference for the social target in both cohorts across all groups (main effect of social target). In addition to examining the presence or absence of significant differences between social and nonsocial targets, we also calculated the social (vs. inanimate) preference index; we found no change in the social preference index among the groups. In the preference for social novelty/social recognition assay, in our first cohort we failed to see the expected main effect of the social target, indicating a lack of preference for the novel social target versus the familiar social target. In our second cohort, we saw the expected main effect of the novel social target versus the familiar social target but no difference among genotypes based on the social novelty preference index. These results suggest homozygous postnatal deletion of *Cul3* in forebrain excitatory neurons does not contribute to robust impairments in social interaction. It further highlights the potential for inconsistent or unexpected results in the preference for social novelty portion of this task even in control or WT groups of mice.

Other studies with early embryonic deletion [17, 18] of *Cul3* (heterozygous) in excitatory neurons report a decrease in sociability and social recognition memory in the 3‐chamber social interaction assay. On the other hand, Morandell et al. [13] saw no changes in sociability or preference for social novelty in delayed postnatal heterozygous *Cul3* deletion mice. It remains unclear whether these differences are due to the use of different mouse models, different developmental timing of deletion, or lack of replicability.

We found robust motor coordination deficits in this mouse model in the accelerating rotarod. Motor coordination impairment on the rotarod was replicable across both cohorts. Interestingly, in constitutive heterozygous *Cul3* mice, motor coordination across Days 2 and 3 was decreased compared to their controls [13]. A small significant decrease in motor coordination was also found on the rotarod in mice with *Cul3* heterozygous deletion in cholinergic neurons [19]. In other studies, however, no effect of heterozygous *Cul3* deletion was observed [16, 17, 18]. Hindlimb clasping, reported in a constitutive heterozygous model [13] and a cell‐type specific model [19], was a phenotype we also observed robustly across both cohorts. In the Morris water maze, swim speed was reduced in Cre‐positive homozygotes, possibly due to impairments in motor coordination, making it difficult to interpret spatial learning and memory in this task. Taken together, our results suggest homozygous *Cul3* deletion in the forebrain of predominantly excitatory neurons postnatally leads to impaired motor coordination.

In the visible platform version of the Morris water maze, a control for visual acuity, we found that *Cul3* homozygotes took longer to find the visible platform compared to controls but eventually swam to the visible platform. Homozygous *Cul3* mice may have decreased visual acuity; alternatively, motor coordination may have caused their prolonged latency to find the visible platform. Future studies with more sensitive measures of visual acuity may be warranted.

In the olfactory food finding assay, which serves as a control for olfaction for sociability tests, we found a slight significant increase in latency to find food in Cre‐positive homozygotes. This may suggest that olfaction was impaired. Given our results on motor coordination, however, it is also possible that *Cul3* homozygotes may lack the motor coordination sufficient to find food as rapidly as controls.

In the marble burying assay, we found a decrease in the number of marbles buried in Cre‐positive *Cul3* homozygotes across both cohorts. One possible speculation is that this could be due to novelty avoidance. Another possibility is that they may avoid digging due to a lack of motivation. In another measure of repetitive behavior, grooming, we found no change in the number of grooming bouts or time spent grooming in *Cul3* homozygotes. Interestingly, in another study, a site‐specific injection of *AAV‐Cre‐CaMKIIα* in the striatum showed increased grooming behavior [17].

Increased locomotor activity was observed in both cohorts. In the first cohort, there was only a trend toward significance, while in our second cohort, this phenotype was robust and statistically significant. Interestingly, *Cul3* constitutive heterozygotes [12] and early embryonic deletion of *Cul3* [17] also showed an increased locomotive phenotype.

In conclusion, *Cul3* deletion postnatally from forebrain excitatory neurons results in robust, internally replicated behavioral differences on multiple behaviors, suggesting altered brain function. That such changes occurred with postnatal deletion of *Cul3* suggests the possibility that some aberrant behaviors may be due to functional brain changes beyond early brain development.

As always, our studies have limitations in direct interpretation. Firstly, *Cul3* mutations in patients with ASD and NDD are constitutive and heterozygous in nature. This mouse model lacks construct validity but gives us insight into how homozygous *Cul3* deletion affects brain function in a cell‐type specific manner and during a specific window postnatally late in neurodevelopment. Further studies exploring the specific roles of other cell types which do not express *CaMKIIα*, inhibitory neurons, nonneuronal cells, among others, at different neurodevelopmental time points may help us glean insight into how *Cul3* deletion affects brain function. Secondly, we also acknowledge that cre‐driven promoters often show mosaicism in their expression even within the cell type in which it is expressed [43, 44, 45, 46]. This fact makes negative findings difficult to interpret as unaffected by loss of *Cul3*. Finally, though we included both sexes in our experiments, we were unable to include sex as a biological factor for our analyses since we are underpowered. Further studies understanding the role of *Cul3* deletion in a sex‐specific manner may help us glean insight into yet undiscovered sex differences.

## Conflicts of Interest

Craig M. Powell acknowledges previous investigator‐initiated research grant support from Novartis and Neuren Inc. Craig M. Powell has had travel reimbursements and honoraria for speaking at Dainippon Sumitomo Pharma Co, Pfizer, Roche, Astra‐Zeneca, and Psychogenics Inc. in the past. These are unrelated to the current study and are disclosed out of an abundance of caution. The other authors declare no conflicts of interest.

## Supporting information


**Figure S1:** (A) At P90, mice that underwent behavior (second cohort) showed no change in weight. (B) In a caged conspecific assay (first cohort), *Cul3* homozygotes spend decreased time in the interaction zone of an object compared to controls. (C) But time spent in the interaction zone with a social target is similar across groups. (D) In the 3‐chamber SI assay, Cre‐positive *Cul3* homozygotes showed a significant preference for chamber A during habituation. (E) *Cul3* homozygotes also showed increase in the time spent in the interaction zone of the social target compared to littermate controls. (F) Social preference index shows increased sociability in Cre‐positive *Cul3* homozygotes. (G) No preference for the novel target was seen when a novel stimulus mouse was introduced across all groups. (H) No change in social novelty preference index across groups. (I) In olfactory food finding assay, Cre‐positive *Cul3* homozygotes show increase in latency to find food. (J) In our second cohort, in the 3‐chamber SI task, no change in preference for chamber across groups.

**Figure S2:** (A) Time spent grooming. (B.)and the number of grooming bouts was similar across groups in our first cohort. (C) In the marble burying assay, Cre‐positive *Cul3* homozygotes show decrease marble burying compared to their littermate controls. (D) Total distance traveled for 20 min showed a trend toward significance in HOM^Cre+^ in our first cohort.

**Figure S3:** (A) Decreased motor coordination and learning in *Cul3* homozygotes on rotarod across 8 trials in our first cohort. (B) Average latency to fall was significantly lower in Cre‐positive *Cul3* homozygotes. (C) On the foot misplacement task (second cohort), the number of forelimb slips. (D) and hindlimb slips was unchanged. (E) Latency to cross the ladder was also unchanged across groups. (F) Grip strength (normalized to weight of the mice) was similar across all genotypes in our second cohort. (G) Representative image of clasping phenotype observed in control WT^Cre+^ and HOM^Cre+^. (H) HOM^Cre+^ show increased hindlimb clasping behavior compared to HET^Cre+^ and WT^Cre+^.

**Figure S4:** (A) In the fear conditioning assay, hippocampus‐dependent contextual fear memory was significantly decreased in *Cul3* homozygotes. (B) with cued fear memory intact across groups in the first cohort. (C) In the Morris water maze (first cohort), latency to platform was significantly decreased in *Cul3* homozygotes across 11 days of training. (D) with average latency to platform per mouse also being significantly decreased in *Cul3* homozygotes. (E) Distance traveled plotted across 11 days of training was significantly increased in *Cul3* homozygotes. (F) with average distance traveled per mouse also significantly increased in *Cul3* homozygotes. (G) Swim speed was significantly decreased in *Cul3* homozygotes across days of training. (H) Average swim speed per mouse was decreased in *Cul3* homozygotes. (I) On the probe trial (Day 12), *Cul3* homozygotes spent significantly less time in the target quadrant compared to their littermate controls and significantly more time in the opposite quadrant.

**Figure S5:** (A) In our second cohort, in the Morris water maze task, latency to platform was significantly increased in Cre‐positive *Cul3* homozygotes across 12 days of training. (B) Average latency to platform for all trials was significantly increased in Cre‐positive *Cul3* homozygotes. (C) Distance traveled across 12 days of training showed a main effect of genotype. (D) When average distance traveled for all trials were plotted, Cre‐positive *Cul3* homozygotes showed an increase in trend toward significance compared to WT controls. (E) Probe trial on Day 8 and (F) Day 14 showed a significant main effect of quadrant. (G) In our first cohort, *Cul3* homozygotes showed a trend toward increase in latency to find the visible platform compared to littermate controls.

**Figure S6:** (A) Percent time spent in open arms was significantly higher in HOM^Cre+^ compared to littermate controls in elevated zero maze (first cohort). (B) However, in our second cohort, percent time spent in open arms was similar across groups. (C) Time in center was decreased significantly in the Cre‐positive homozygotes compared to littermate controls in the open field (first cohort). (D) Time spent in the center was similar across groups in our second cohort. (E) In the dark/light assay, no change in the latency to enter light chamber was observed across groups (first cohort). (F) Time spent in the light chamber was also unchanged across groups. (G) In our second cohort, no change in the latency to enter light chamber. (H) and time spent in the light chamber was comparable across genotypes.

**Figure S7:** (A) In the nest building assay, *Cul3* homozygotes built nests with significant decrease in height and (B) no change in the width in our first cohort. (C) In our second cohort, no change in the height or (D) the width of the nest across groups.

**Table S1:** Statistical summary of behavioral and biochemical assays.

## Data Availability

The data that support the findings of this study are available from the corresponding author upon reasonable request.
