# Supplementary figures and images for: Cul3 Postnatal Homozygous Deletion in Forebrain Results in Behavioral Differences
Source: Genes Brain Behav. 2025 Oct 20;24(5):e70039. doi: 10.1111/gbb.70039 (PMC12536218; doi:10.1111/gbb.70039)

**A**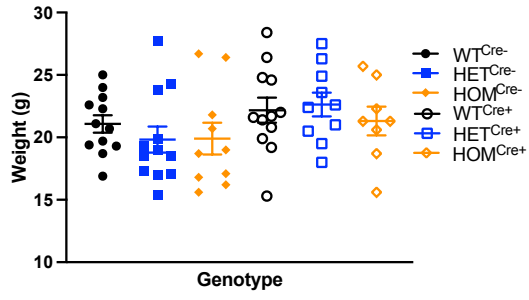**B**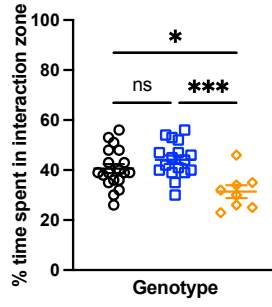**C**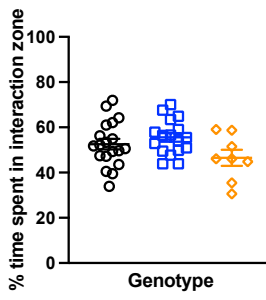**D**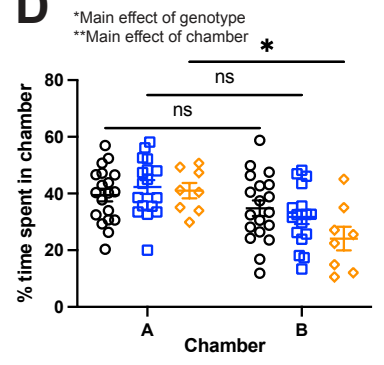**E**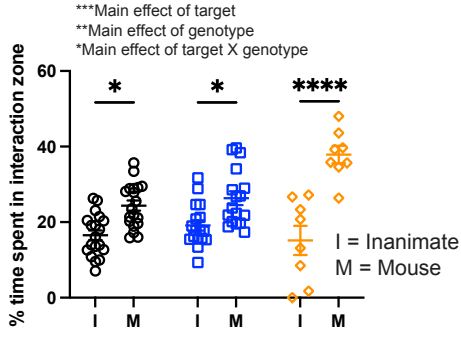**F**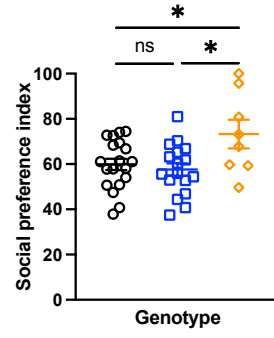**G**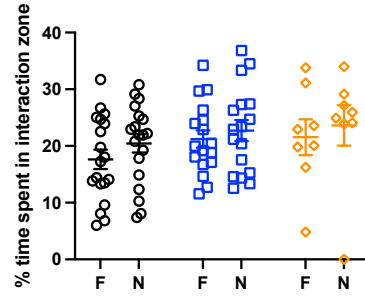**H**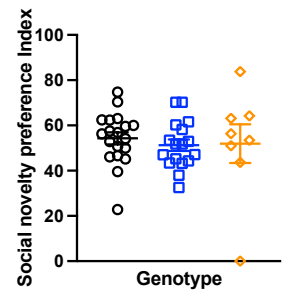**I**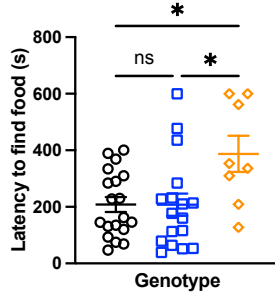**J**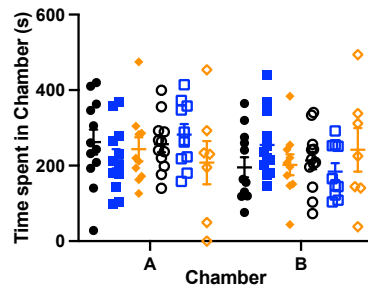

Supplement: Supplementary file 1 — Figure S1: (A) At P90, mice that underwent behavior (second cohort) showed no change in weight. (B) In a caged conspecific assay (first cohort), Cul3 homozygotes spend decreased time in the interaction zone of an object compared to controls. (C) But time spent in the interaction zone with a social target is similar across groups. (D) In the 3‐chamber SI assay, Cre‐positive Cul3 homozygotes showed a significant preference for chamber A during habituation. (E) Cul3 homozygotes also showed increase in the time spent in the interaction zone of the social target compared to littermate controls. (F) Social preference index shows increased sociability in Cre‐positive Cul3 homozygotes. (G) No preference for the novel target was seen when a novel stimulus mouse was introduced across all groups. (H) No change in social novelty preference index across groups. (I) In olfactory food finding assay, Cre‐positive Cul3 homozygotes show increase in latency to find food. (J) In our second cohort, in the 3‐chamber SI task, no change in preference for chamber across groups. [file GBB-24-e70039-s001.pdf]

**A**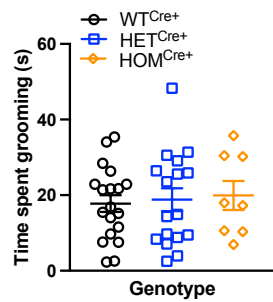**B**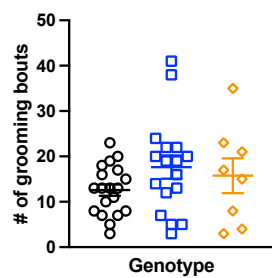**C**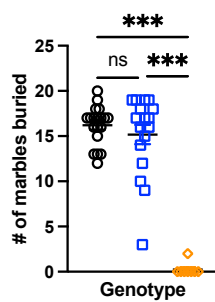**D**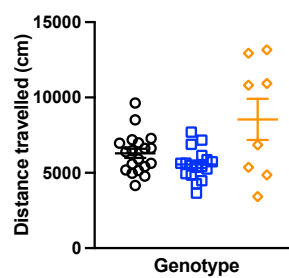

Supplement: Supplementary file 2 — Figure S2: (A) Time spent grooming. (B.)and the number of grooming bouts was similar across groups in our first cohort. (C) In the marble burying assay, Cre‐positive Cul3 homozygotes show decrease marble burying compared to their littermate controls. (D) Total distance traveled for 20 min showed a trend toward significance in HOMCre+ in our first cohort. [file GBB-24-e70039-s005.pdf]

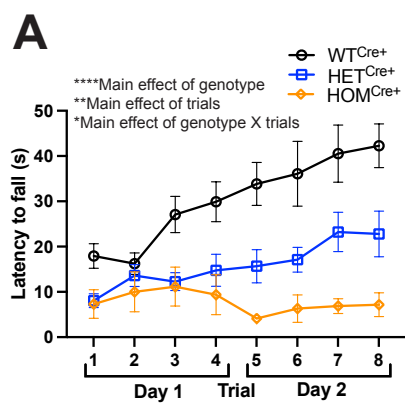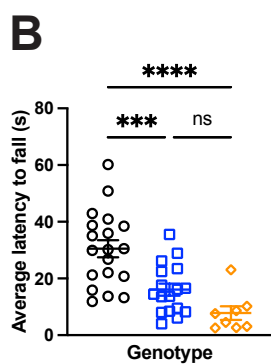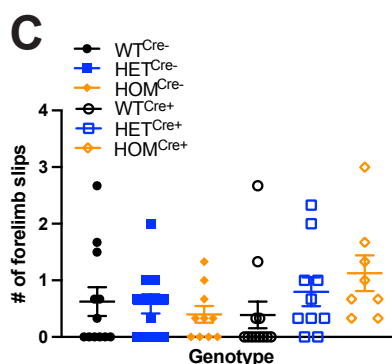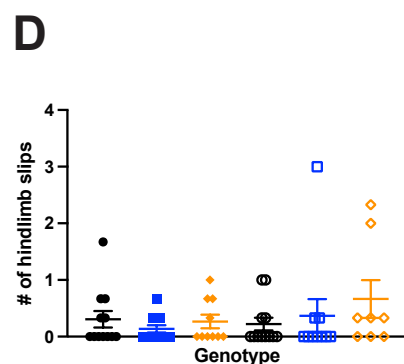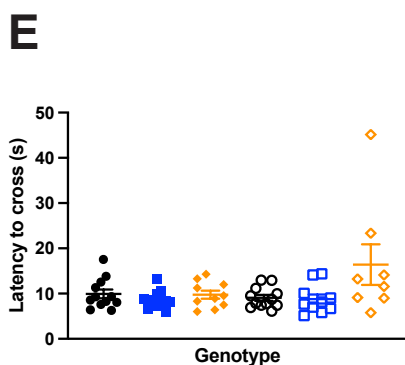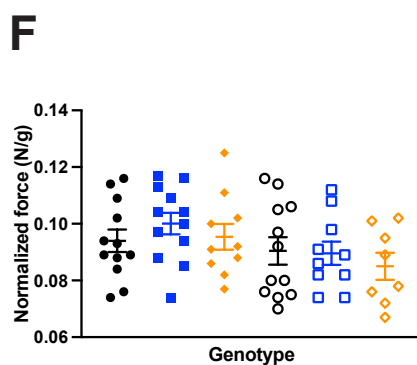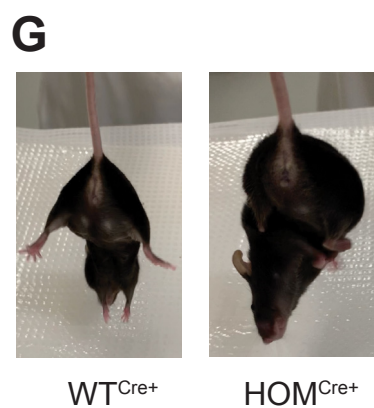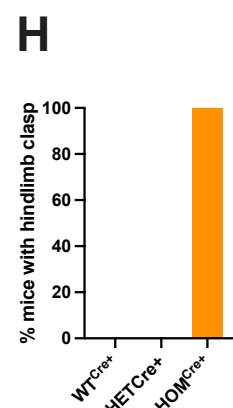

Supplement: Supplementary file 3 — Figure S3: (A) Decreased motor coordination and learning in Cul3 homozygotes on rotarod across 8 trials in our first cohort. (B) Average latency to fall was significantly lower in Cre‐positive Cul3 homozygotes. (C) On the foot misplacement task (second cohort), the number of forelimb slips. (D) and hindlimb slips was unchanged. (E) Latency to cross the ladder was also unchanged across groups. (F) Grip strength (normalized to weight of the mice) was similar across all genotypes in our second cohort. (G) Representative image of clasping phenotype observed in control WTCre+ and HOMCre+. (H) HOMCre+ show increased hindlimb clasping behavior compared to HETCre+ and WTCre+. [file GBB-24-e70039-s006.pdf]

**A**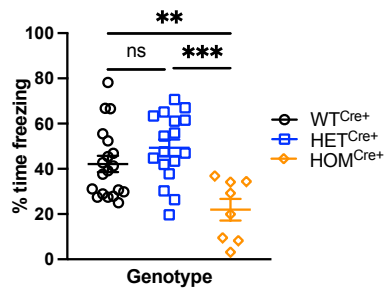**B**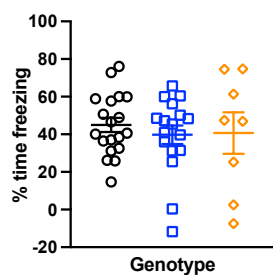**C**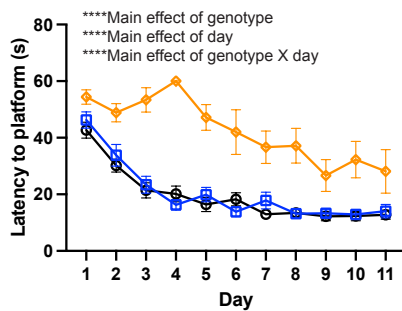**D**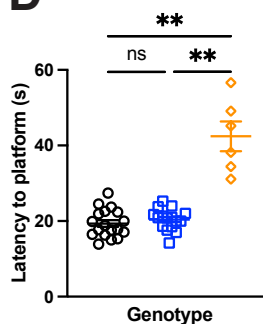**E**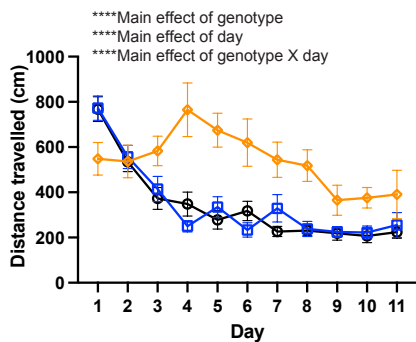**F**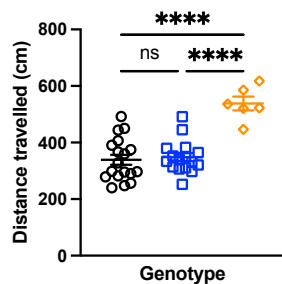**G**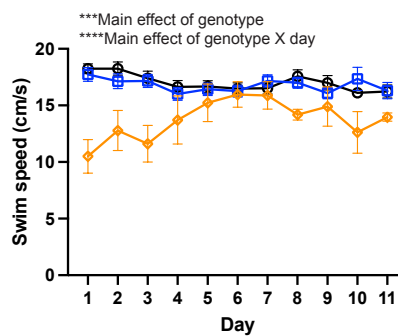**H**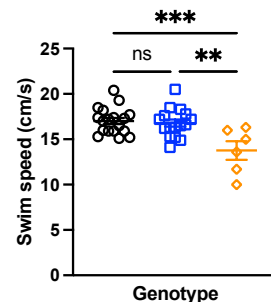**I**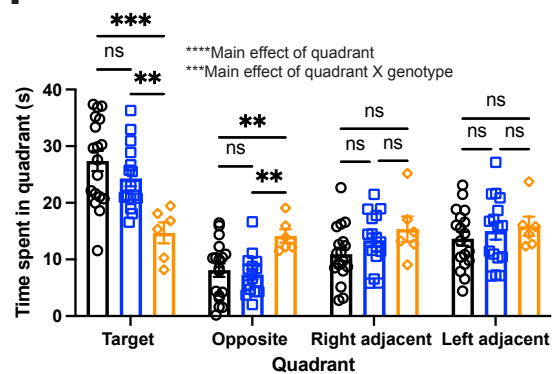

Supplement: Supplementary file 4 — Figure S4: (A) In the fear conditioning assay, hippocampus‐dependent contextual fear memory was significantly decreased in Cul3 homozygotes. (B) with cued fear memory intact across groups in the first cohort. (C) In the Morris water maze (first cohort), latency to platform was significantly decreased in Cul3 homozygotes across 11 days of training. (D) with average latency to platform per mouse also being significantly decreased in Cul3 homozygotes. (E) Distance traveled plotted across 11 days of training was significantly increased in Cul3 homozygotes. (F) with average distance traveled per mouse also significantly increased in Cul3 homozygotes. (G) Swim speed was significantly decreased in Cul3 homozygotes across days of training. (H) Average swim speed per mouse was decreased in Cul3 homozygotes. (I) On the probe trial (Day 12), Cul3 homozygotes spent significantly less time in the target quadrant compared to their littermate controls and significantly more time in the opposite quadrant. [file GBB-24-e70039-s004.pdf]

**A**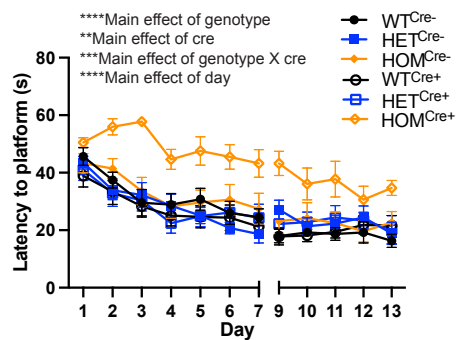**B**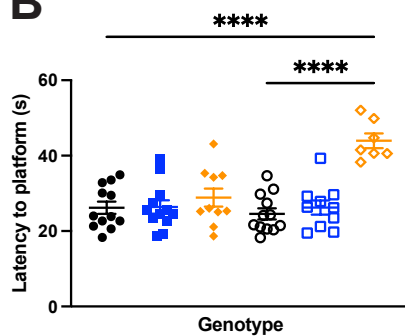**C**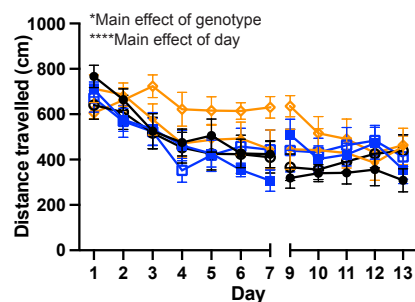**D**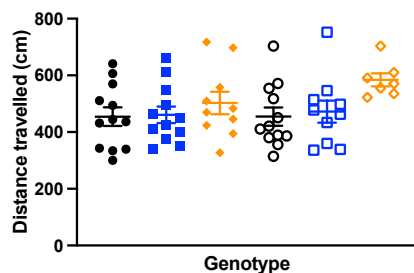**E**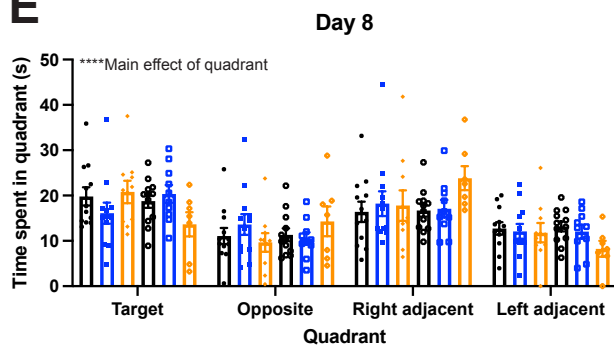**F**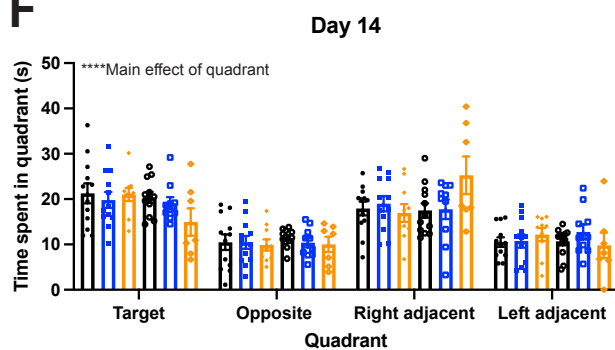**G**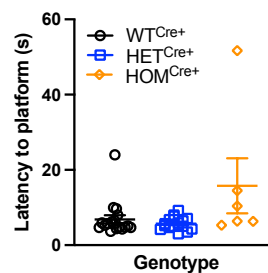

Supplement: Supplementary file 5 — Figure S5: (A) In our second cohort, in the Morris water maze task, latency to platform was significantly increased in Cre‐positive Cul3 homozygotes across 12 days of training. (B) Average latency to platform for all trials was significantly increased in Cre‐positive Cul3 homozygotes. (C) Distance traveled across 12 days of training showed a main effect of genotype. (D) When average distance traveled for all trials were plotted, Cre‐positive Cul3 homozygotes showed an increase in trend toward significance compared to WT controls. (E) Probe trial on Day 8 and (F) Day 14 showed a significant main effect of quadrant. (G) In our first cohort, Cul3 homozygotes showed a trend toward increase in latency to find the visible platform compared to littermate controls. [file GBB-24-e70039-s002.pdf]

**A**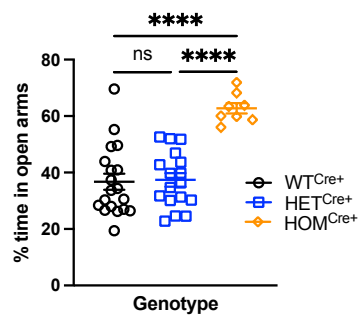**B**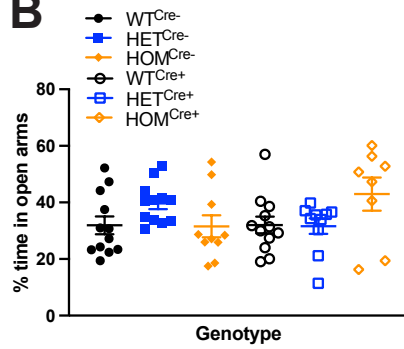**C**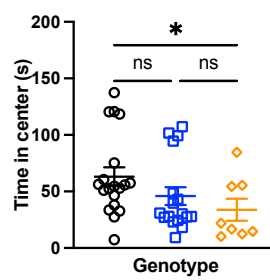**D**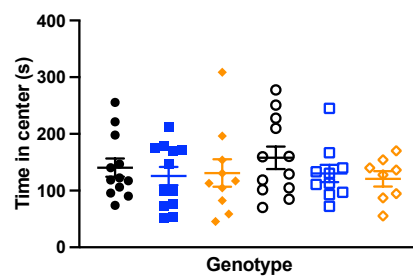**E**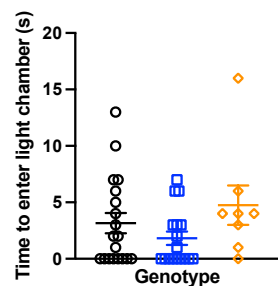**F**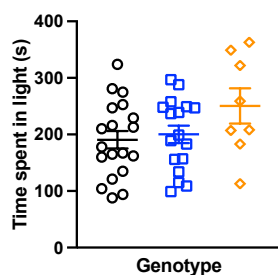**G**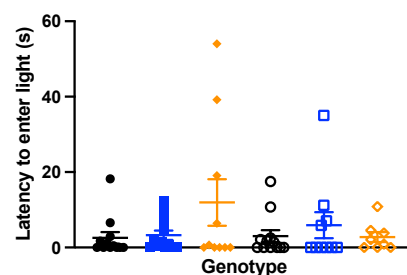**H**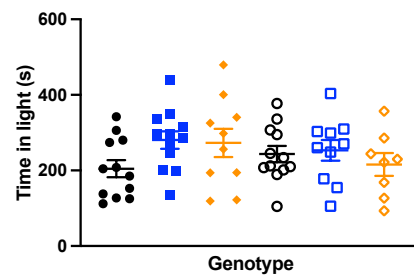

Supplement: Supplementary file 6 — Figure S6: (A) Percent time spent in open arms was significantly higher in HOMCre+ compared to littermate controls in elevated zero maze (first cohort). (B) However, in our second cohort, percent time spent in open arms was similar across groups. (C) Time in center was decreased significantly in the Cre‐positive homozygotes compared to littermate controls in the open field (first cohort). (D) Time spent in the center was similar across groups in our second cohort. (E) In the dark/light assay, no change in the latency to enter light chamber was observed across groups (first cohort). (F) Time spent in the light chamber was also unchanged across groups. (G) In our second cohort, no change in the latency to enter light chamber. (H) and time spent in the light chamber was comparable across genotypes. [file GBB-24-e70039-s003.pdf]

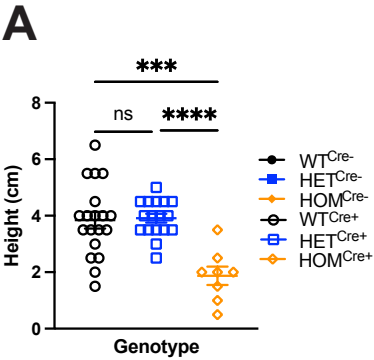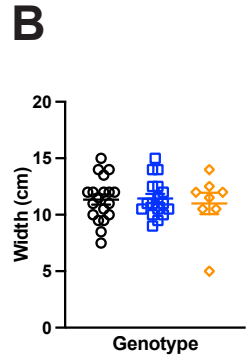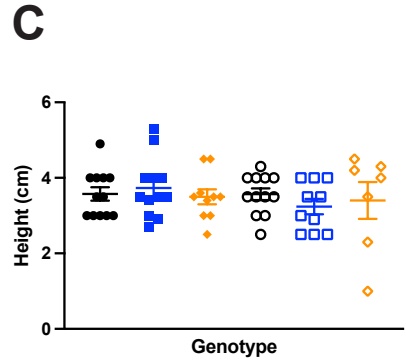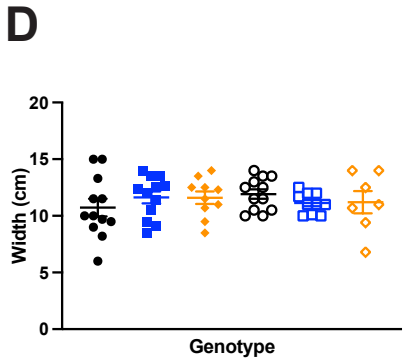

Supplement: Supplementary file 7 — Figure S7: (A) In the nest building assay, Cul3 homozygotes built nests with significant decrease in height and (B) no change in the width in our first cohort. (C) In our second cohort, no change in the height or (D) the width of the nest across groups. [file GBB-24-e70039-s007.pdf]
